# Supplementary material for: Transcriptome and Metabolome Analysis of Isoquinoline Alkaloid Biosynthesis of Coptis chinensis in Different Years
Source: Genes (Basel). 2023 Dec 18;14(12):2232. doi: 10.3390/genes14122232 (PMC10742649; doi:10.3390/genes14122232)
Supplement: Supplementary file 1 [file genes-14-02232-s001.zip › Supplementary Figures Table S1.pdf]

Supplementary Table S1 Samples sequencing data evaluation

Sample	Raw Reads	Clean Reads	Clean Base(G)	Q20(%)	Q30(%)	GC Content(%)
Cch2-1	43219666	41432808	6.21	97.63	93.16	42.72
Cch2-2	46668210	44663620	6.7	97.63	93.14	42.35
Cch2-3	47915720	45880232	6.88	97.67	93.25	42.7
Cch4-1	48929294	46932992	7.04	97.67	93.24	42.6
Cch4-2	63273278	61381392	9.21	98.16	94.37	42.5
Cch4-3	42272492	40405586	6.06	97.67	93.26	42.59

Note: Cch2: 2-year *Coptis. chinensis*, Cch4: 4-year *Coptis. chinensis*

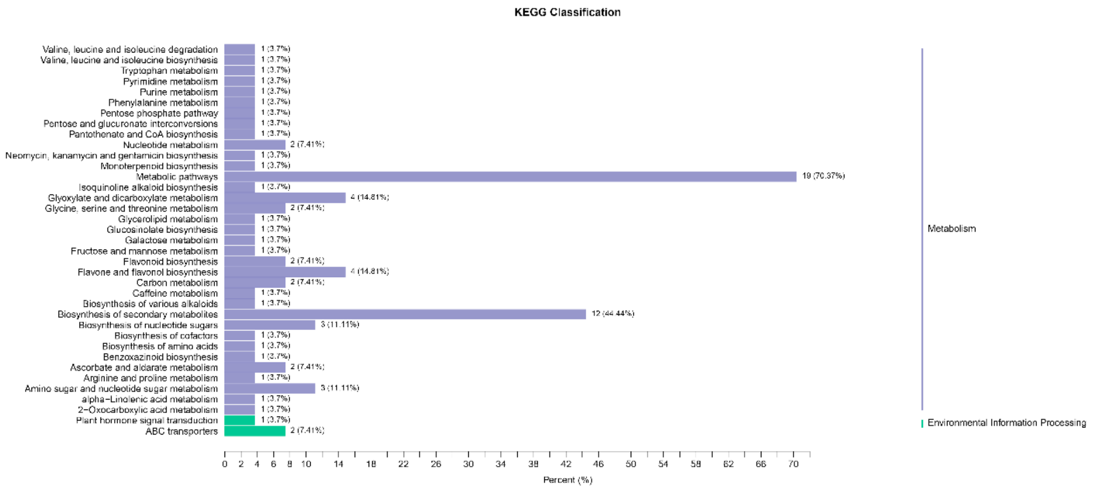

Figure S1 Annotation of differentially metabolites in *C. chinensis* in KEGG

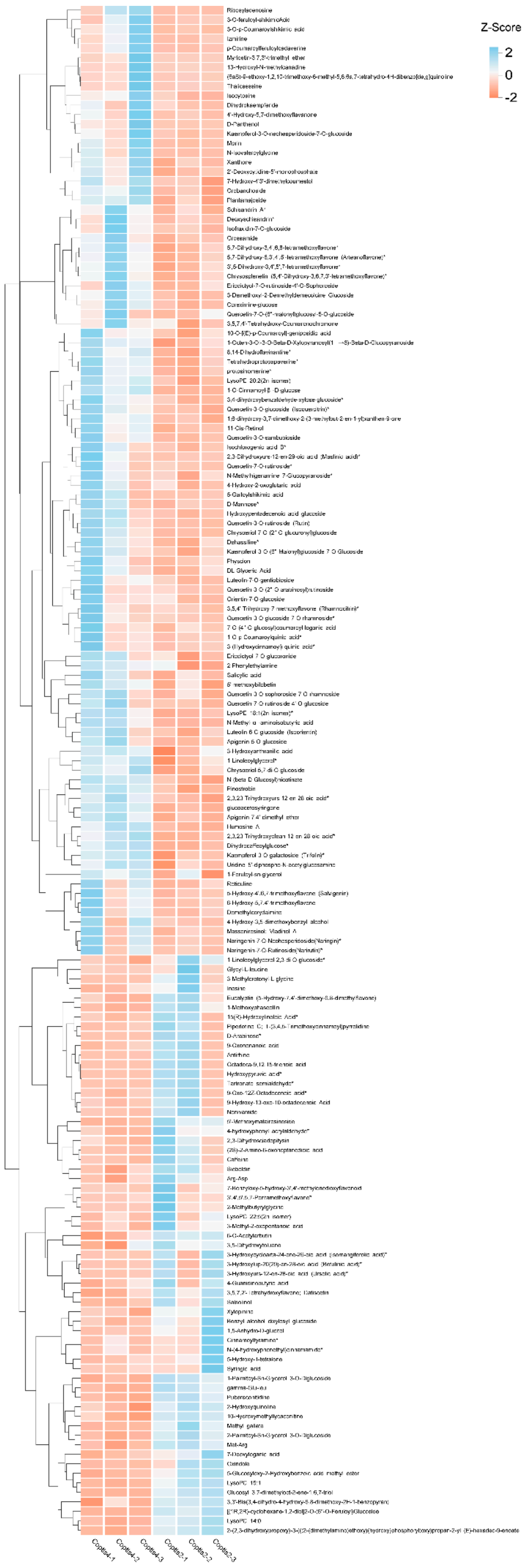
Z-Score
2
0
-2
Coptis4-1
Coptis4-2
Coptis4-3
Coptis2-1
Coptis2-2
Coptis2-3

Figure S2 The content heatmap of all DEMs. Each column represents a biological repeat, and each row represents a metabolite.

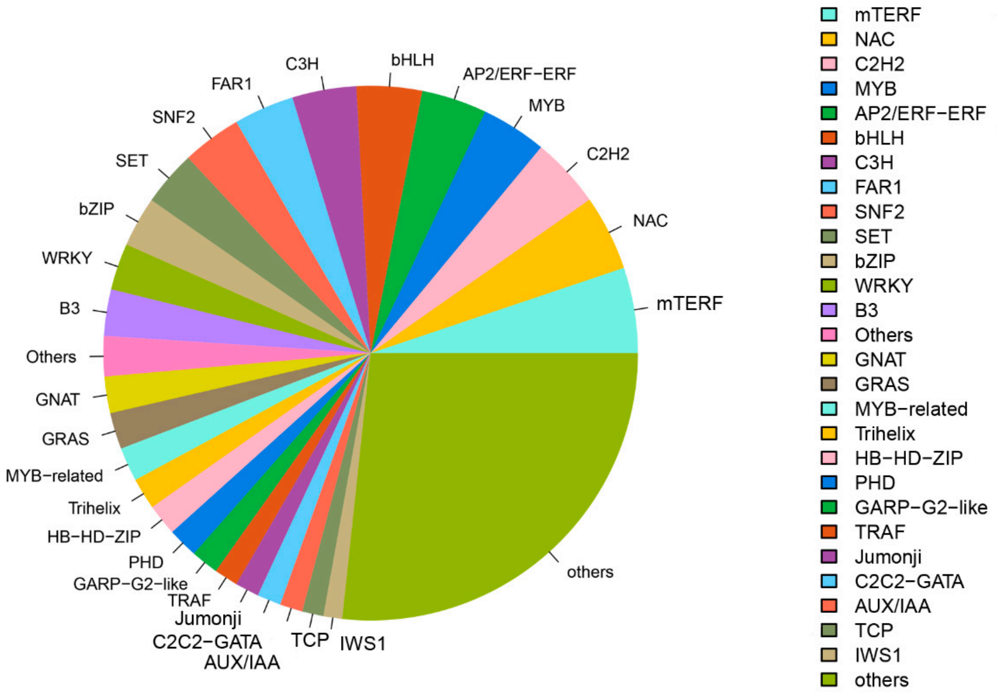

Figure S3 Classification of differentially expressed transcription factors in *C. chinensis.*

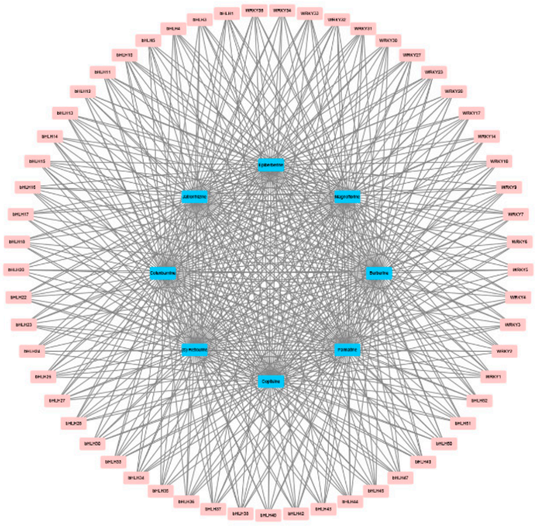

Figure S4. Correlation network diagram between WRKYs, bHLHs and important alkaloids. Blue boxes are metabolites, pink boxes are genes, and the thickness of the line represents the high or low correlation.
