# Supplementary material for: Transcriptome and Metabolome Analysis of Isoquinoline Alkaloid Biosynthesis of Coptis chinensis in Different Years
Source: Genes (Basel). 2023 Dec 18;14(12):2232. doi: 10.3390/genes14122232 (PMC10742649; doi:10.3390/genes14122232)
Supplement: Supplementary file 1 [file genes-14-02232-s001.zip › Supplementary Table S8 Reference sequences for different species.pdf]

OMT

>St6OMT1

MAAENQKQHQAAQAKLWSFIYGYSESLVLKSAVQLDIPLIIHNHGKPISLSDLACQIPSPSG
AVDHDRLYRIMRYLVHMKLFTKESVEGEDRYGLAPPAKYLVKGWEKSMVPSILCVTDKE
FMSPWYHLKEALSNDSTTAFEKALGMNIWDYMSKNPKKNIIFNEAMACETSQIMSALVSE
CKDMFEGIKSLVDVGGGNGTAVRHIAKAFPHIICTVYDLPHVAADSPTYPEVSRVEGDMFK
FIPKADAILMKCILHDWADEECIKILKRCKEALPSNGGKIILVEVVLNVFSEHPYTKIRMLS
DLDMMLNCGGKERTEEEWKKLIYAAGFSRHNIRQVDFTQSVIEVYP

>St6OMT2

MEEEMKAQAQVWRYVYGFAESLTLKCAIQLNIPDILHHHNRPMTLSELATHLPLPSVNQD
RLYRVMRYLVHMGLFDLLDSPDSPKYSLNSASKLLVKGQDKSLASFALLQYYEMDAWHH
LSAAVEGAATPWEKCHGVDYREYFAKDAVANQLLSDAMTSHTSMVTEALVEGCKKGGV
LHGVRSFVDVGGSTGVAARAVAKAFPGIKCSVFDLPHVIATAPECPEVTRIQGDMFVSIPET
DVVFMKSVLHDWGDEDCVKILKKCKEAIAGKEGGKVVILDIVLDAESSYEFKGARLGME
MDMLVTVGGKERSEAEWQKLFKDAGYSRYKITPIVAIESVIEVFP

>St6OMT3

MEDIGKKRETAISENDYDDEEQRAEIDMWRYIFGFSEMAAVKCAIELGIAEAMEAHGRPM
SLSELATALKCSSSYLYRILRFLVHLRIFKEKCSNAANGTKLYTLSPLGRLLMKQGEHGMA
ALVLLESSPVMLAPWHALSGRVRNNERPAFEAAHGEDVWAFAAGNPNHSMLINEAMAC
DARMVVPAIVNGCADVFDGVDSVVDVGGGDGTTLGMLVKAFPWIKGINFDLPHVLSVAP
GRNGVEHLGGDMFKAVPQADAAFLMWVLHDWCDEECIEILKRCREAIPKERGKVIIVDA
VIEEADDEQEKEH
GDENYKLKGAKLMLDMVMMAHTNRGKERTQEEWQYVLDKAGFSRFVVRPIRAVQSVI
VAYC

>St6OMT4

MSTIAGEEQEQEMRWQAQVWNHISGGFVNSIVLKCAADLSLFDTIQAIHTKQGKPVTPST
LVRTSSLSSINPHKLFRLLRFLAHMKLLTIHAFDDVGDGGNEVGFSLTDLAKLMLRRNEKN
LVDWVIGVPDEPAINAWHQLGNCVSYPSEGPTAFEEEHGKTLWEFARDNPEMNKVFNDA
MACDTRLVMPAFIEGCKDVLKGISSLVDVGGGTGTAMSYVIKAFPHVKCTVYDLPHVITT
SPEYPGIELIGGDMFQSVPQADAVLLKFMLHNWQNEECVKILKKCKEAVPKEKGKVIIVD
VVIDQDEEDYDVTHIRLSLDMDMMGMSGGRERTKEEWRVLMKMAGFSHYEIIPIFAVQS
VIVAYP

>AthOMT

MGSTAETQLTPVQVTDDEAALFAMQLASASVLPMALKSALELDLLEIMAKNGSPMSPTEI
ASKLPTKNPEAPVMLDRILRLLTSYSVLTCSNRKLSGDGVERIYGLGPVCKYLTKNEDGVS
IAALCLMNQDKVLMESWYHLKDAILDGGIPFNKAYGMSAFEYHGTDPRFNKVFNNGMS
NHSTITMKKILETYKGFEGLTSLVDVGGGIGATLKMIVSKYPNLKGINFNLPHVIEDAPSHP
GIEHVGGDMFVSVPKGDAIFMKWICHDWSDEHCVKFLKNCYESLPEDGKVILAECILPET
PDSSLSTKQVVHVDCIMLAHNPGGKERTEKEFEALAKASGFKGIKVVCD
AFGVNLIELLKKL

>CanODOMT

MDSTNQNLTQTEDEAFLFAMQLASASVLPMVLKSALELDLLEIMAKAGPGAAISPSELAA
QLPTKNPEAPVMLDRMLRLLATYSVLNCTLRTLPDGRVERLYSLAPVCKLLTKNADGVSV
APLLLMNQDKVLMESWYHLTDAVLDGGVPFNKAYGMTAFEYHGTDPRFNKVFNRGMS

DHSTMTMKKILEDYKGFEGLNSIVDVGGGTGATVNMIVSKYPSIKGINFDLSHVIEDAPAY
PGVEHVGRDMFVSVPKADAIFMKWICHDWSDEHCLKFLKNCYEALPANGKVLVAECILP
ETPDTSAATKNAVHVDIVMLAHNPGGKERTEKEFEALAKGAGFTGFRRACCAYQTWVME
FHK
>CamCaOMT
MLFAMQLACASVLPMVLKSAIELDLLEIIRGQDTCMSPTEIASHLPTTNPDAPAMVDRILR
LLSCYSVVTCSVRSVDDQRVYGLAPVCKYLTKNQDGVSIAALCLMNQDKVLMESWYHL
KDAVLDGGIPFNKAYGMSSFEYHGTDPRFNKVFNRGMSDHSTITMKKVFQTYQGFQGLT
SLVDVGGGTGATLTMILSKYPTIRCINFDLPHVIEDAPEYPGIEHVGGDMFVSVPKGDAIFM
KWICHDWSDEHCLKLLKNCYDALPNNGKVILAECILPEVPDSSLATKGVVHIDVITVAHN
PGGKERTEKEFEALAKAAGFQGFQVFCNAFNTYIIEFSKKICN
>Cam3'FOMT
MLFAMQLASASVLPMVLKSAIELDLLEIIASQDTCMSPTEIASHLPTTNPHAPTMIDRILRL
LSSYSIVT
CSVRSVDDQRVYSPAPVCKYLTKNQDGVSIAALCVAAQDKVLMECWYHMKDAVLDGGI
PFNKAYGMPIFDYFAKDLGSNKVFNKGMSDFSSMIIKKILETYKGFQGLTSLVDVGGGTG
ATLTKILSKYPTIRGINFDLPHVIQDAPEYPGIEHVGGDMFVSVPKGDAIFMKWICHDWNE
EQCLKLLKNCYDALPNNGKVIVAEYILPVVPDSSLASKLSVTADVMIVTQNSGGKERTEK
EFEALAKAAGFQGFQVFCNAFTIYIIEFSKNISN
>CbrCaOMT
MGSTGNAETQLTPTHVSDEEANLFAMQLASASVLPMVLKAAIELDVLEIMAKSIPHGSGA
YISPAEIAAQLPTTNPDAPVMLDRVLRLLASYSVVTCSLRELPDGKVERLYGLAPVCKFLT
KNEDGVSLAPLCLMNQDKVLMESWYYLKDAILDGGIPFNKAYGMTAFEYHGTDPRFNK
VFNRGMSDHSTITMKKIFEMYTGFEALNTIVDVGGGTGAVLSMIVAKYPSIKGINFDLPHV
IEDAPIYPGVEHVGGDMFVSVPKGDAIFMKWICHDWSDEHCLKFLKNCYAALPEHGKVI
VAECILPLSPDPSLATKGVIHIDAIMLAHNPGGKERTEKEFEALAIGAGFKGFKVACCAFNT
YVMEFLKTA
>CbrIeOMT
MGSTGNAEIQIIPTHSSDEEANLFAMQLASAAVLPMALKAAIELDVLEIMAKSVPPSGYISP
AEIAAQLP
TTNPEAPVMLDRVLRLLASYSVVTYTLRELPSGKVERLYGLAPVCKFLTKNEDGVSLAPF
LLTATDKVLL
EPWFYLKDAILEGGIPFNKAYGMNEFDYHGTDHRFNKVFNKGMSSNSTITMKKILEMYN
GFEGLTTIVDVGGGTGAVASMIVAKYPSINAINFDLPHVIQDAPAFSGVEHLGGDMFDGVP
KGDAIFIKWICHDWSDEHCLKLLKNCYAALPDHGKVIVAEYILPPSPDPSIATKVVIHTDAL
MLAYNPGGKERTEKEFQALAMASGFRGFKVASCAFNTYVMEFLKT
>Cch6OMT2
MVKKDNLSSQAKLWNFIYGFAESLVLKCAVQLDLANIIHNNGTSMTLSELSSRLPSQPVNE
DALYRVMRYLVHMKLFTKASIDGELRYGLAPPAKFLVKGWDKCMVGSILAITDKDFMAP
WHYLKDGLAGESGTAFEKALGMNIWGYMAEHPEKNQLFNEAMANDSRLIMSALVKECG
NIFNGITTLVDVGGGTGTAVRNIANAFPHIKCTVYDLPHVIADSPGYSEVHCVAGDMFKFIP
KADAIMMKCILHDWDDKECIEILKRCKEAVPIEGGKVIIVDIVLNVQSEHPYTKMRLTLDL
DMMLNTGGKERTEEEWKNLIHDAGYKGHKITQITAVQSVIEAYPY
>Cja6OMT

MEVKKDNLSSQAKLWNFIYGFAESLVLKCAVQLDLANIIHNSGTSMTLSELSSRLPSQPVN
EDALYRVMRYLVHMKLFTKASIDGELRYGLAPPAKYLVKGWDKCMVGSILAITDKDFMA
PWHYLKDGLSGESGTAFEKALGTNIWGYMAEHPEKNQLFNEAMANDSRLIMSALVKEC
GNIFNGITTLVDVGGGTGTAVRNIANAFPHIKCTVYDLPHVIADSPGYSEVHCVAGDMFKF
IPKADAIMMKCILHDWDDKECIEILKRCKEAVPVKGGKVIIVDIVLNVQSEHPYTKMRLTL
DLDMMLNTGGKERTEEEWKKLIHDAGYKGHKITQITAVQSVIEAYPY

>Cja4'OMT

MAFHGKDDVLDIKAQAHVWKIIYGFADSLVLRCAVELGIVDIIDNNNQPMALADLASKLP
VSDVNCDNLYRILRYLVKMEILRVEKSDDGQKKYALEPIATLLSRNAKRSMVPMILGMTQ
KDFMTPWHSMKDGLSDNGTAFEKAMGMTIWEYLEGHPDQSQLFNEGMAGETRLLTSSLI
SGSRDMFQGIDSLVDVGGGNGTTVKAISDAFPHIKCTLFDLPHVIANSYDLPNIERIGGDM
FKSVPSAQAIILKLILHDWNDEDSIKILKQCRNAVPKDGGKVIIVDVALDEESDHELSSTRLI
LDIDMLVNTGGKERTKEVWEKIVKSAGFSGCKIRHIAAIQSVIEVFP

>CjaCoOMT

MDTPNTFQNDDEIKAQAQVWKHMFGFAETIMLRSTVSLGIPDIIHNNGPVTLSQLVTHLPL
KSTSIDRFHHFMRYLVHMQLFTISTDQITKEDKYELTPASKLLVHGHQKSLAPYVMLQTHP
EEFSVWSHVINVLDGKKPYWESNDTSMYEKTEGDPEINEILNDAMTSHSTFMLPALVSGL
MKENVLDGVASIVDVGGNSGVVAKGIVDAFPHVKCSVMDLNHVIERVIKNPKLDYVAGD
MFTSIPNADAILLKSTLHNYEDDDCIKILNIAKEALPST
GGKVILVEIVVDTENLPLFTSARLSMGMDMMLMSGKERTKKEWEDLLRKANFTSHQVIPI
MAIESIIVAYS

>CjaSOMT

MCTSLSELKCPVFSTKRKLLLEFALRTSVDMAAQEGVNYLSGLGLSRLICLPMALRAAIEL
NVFEIISQA
GPDAQLSPSDIVAKIPTKNPSAAISLDRILRMLGASSILSVSTTKSGRVYGLNEESRCLVASE
DKVSVVP
MLLFTSDKAVVESFYNIKDVVLEEGVIPFDRTHGMDFFQYAGKEERVNKSFNQAMGAGST
IAFDEVFKVYKGFDNLKELVDVGGGIGTSLSNIVAKHPHIRGINFELPHVIGDAPDYPGVEH
VPGDMFEGVPNAQNILLKWVLHDWDDDRSIKILKNCWKALPENGTVIVIEFVLPQVLGN
NAESFNALTPDLLMMALNPGGKERTTIEFDGLAKAAGFAETKFFPISQGLHVMEFHKINC

>Cmi6OMT

MEVQKGDQAAQAKIWRLIYGFSESLVLRCAVELGIADIIHEHGDTISINQLASKIPVNSVNV
DHLYRIMRFLVHMKLFSIQSVKGELRYGLEPAARCLVKGWDKCMVPSILSITDKDFMAPW
HYIKESLGKDSPTAFERALGMNIWDYMADNPKKNQLFNEGMACDSRLLTSALIQDCKEM
FEGILSLVDVGGGTGTALRAIAKAFPHLKCTVYDLPHVIADSPDYPEVARVPGDMFKSIPS
ADAIMMKCILHDWDDQECIKILKKCREAVPPKGGKVIIVDIVLDVESDCHYSNMRRCMDL
DMMLNTGGKERTEEEWKKLIYDAGFTGYKIKSISAVQSVIEAYPY

>CroCaOMT

MGSANPDNKNSMTKEEEEACLSAMRLASASVLPMVLKSAIELDLLELIKKSGPGAYVSPS
ELAAQLPTQNPDAPVMLDRILRLLASYSVLNCTLKDLPDGGIERLYSLAPVCKFLTKNEDG
VSMAALLLMNQDKVLMESWYHLKDAVLEGGIPFNKAYGMTAFEYHGKDPRFNKVFNQ
GMSNHSTIIMKKILEIYQGFQGLKTVVDVGGGTGATLNMIVSKYPSIKGINFDLPHVIEDAP
SYPGVDHVGGDMFVSVPKGDAIFMKWICHDWSDAHCLKFLKNCHEALPENGKVILAEC
LLPEAPDSTLSTQNTVHVDVIMLAHNPGGKERTEKEFEALAKGAGFRGFIKVCCAYNSWI

MELLK
>Eca4'OMT
MGLEFNEEVDIKAQAHLWNIIYGFADSLVLRSAVELGIADIIKNNNGSITVSELASKLPISNV
NSDNLYR
VLRYLVHMGILKETKSTINGGEIKKLYSLEPVGSLLVKDAERNMVPIVLGMTQQDFMIPW
HYIKEGLGEGSTAFEKGMGMTLWEYLEGHPEQGHLFNVGMEGETRLLTKTLIESCKDTFE
GLSSLVDVGGGNGTTIKAISEAFPHIKCSLYDLPHVVADSHDLPNIEKIPGDIFKFIPNAQAIL
LKLILHDWSDEDSVKILKKCREAVPQDTGRVIIVDVALEEESEHPLTKTRLVLDVDMLVNT
GGRERSEDDWAKLLKLAGFRTHKIRHIAAVQSVIEAFP
>Eca7OMT
MDEEIILGQADICKYMYGFVDSMTLRCVVELGIPDIIHSHGRPITLTEILNGIPNLSSSFDINY
LQGIMT
ILVRRRVFAVHKFDPKDGTNLTEIRYGLTPSSKCLLKDSKFNLAPFVLLETHPWITDPWNYL
GKCVQEGGSGFVKAHGSDVFKFGSDHPEFFKLFYDGMECSTKVLVQVVLDKYQQVFKD
VKSIVDVGGGTGMMISEIVKNHPHIKGINFDLPHVVAEAPDYPGVEHVGGDMFVEIPQAD
AITMKGILHDWNDDACVKILENCKKAIPKNGKVIIIDCVLNPDGDDLFDDIKVVSDLGMR
VHCSDGKERTEAEWEKLLKKGGFPRYKITHVVTVQSMIEAYPE
>EcaOMT
MEMEGADKIDQATQAKIWKHIYGFAESLILKSAVELEIADTIHNHGKPMTLSELASKLPVQ
PVDLDRLYRVMRYLVHMKLFKKQEDEDEESIKYSLLPPAKFLIKGWEKSMVSAILLINDK
DFMSPWHHLKEGLVNNSNAFDKALGKSIWDYMSANPDKNKVFNEGMACDSRLVSSALV
NECGNIFQGIQTLVDVGGGTGTAVKAVSKAFPHIKCSIYDLPHVISDSPDIPNIVKIEGEMFK
SIPSADAILMKCILHDWNDDECIEILKKCKEAVPKEGGKVIIVDIVLDMESKHPYSKVRLTL
DLDMMLNTEGKERTEEEWKKLIDGAGFASYKITQISALQSVIEAFPY
>EglCaOMT
ANLFAMQLASASVLPMVLKAAIELDLLEIMAKAGPGAFLSPGEVAAQLPTQNPEAPVMLD
RISRLLASYSVLTCTLRNLPDGKVERLYGLAPVCKFLVKNEDGVSIAALNLMNQDKILMES
WYYLKDAVLEGGIPFNKAYGMTAFEYHGTDPRFNKIFNRGMSDHSTITMKKILETYKGFE
GLETVVDVGGGTGAVLSMIVAKYPSMKGINFDLPHVIEDAPPLPGVKHVGGDMFVSVPK
GDAIFMKWICHDWSDDHCAKFLKNCYDALPNNGKVIVAECVLPVYPDTSLATKNVIHIDC
IMLAHNPGGKER
>EguOMT
MGSTGSETQMTPTQVSDEEANLFAMQLASASVLPMVLKAAIELDLLEIMAKAGPGAFLSP
GEVAAQLPTQNPEAPVMLDRIFRLLASYSVLTCTLRNLPDGKVERLYGLAPVCKFLVKNE
DGVSIAALNLMNQDKILMESWYYLKDAVLEGGIPFNKAYGMTAFEYHGTDPRFNKIFNR
GMSDHSTITMKKILETYKGFEGLETVVDVGGGTGAVLSMIVAKYPSMKGINFDLPHVIED
APPLPGVKHVGGDMFVSVPKGDAIFMKWICHDWSDDHCAKFLKNCYDALPNIGKVIVAE
CVLPVYPDTSLATKNVIHIDCIMLAHNPGGKERTQKEFETLAKGAGFQGFQVMCCAFGTH
VMEFLKTA
>GecOMT
MSDSYSTKDNNLFSTSSEQTEDGACLSAMRLVTNLVYPAVLNAAIDLNLFEIIAKATPPGAF
MSASEIASKLPLPTQHSDLPNRLDRMLRLLASYSVLTCATRSTERVYGLSQVGKYLVPDGS
RGYLASFTTFLCYPALMNVWLNFKEAVVDEDIDLFKKLHGVSKYEYMETDPKMNHIFNK
SMADVCATEMKRILQIYKGFEGISTLVDVGGGNGQNLKMIISKYPLIKGINFDLPQVIENAP

PIPGIELVGGDMFASVPQGDAMILKAVCHNWSDEKCLEFLSNCHKALSPNGKVIVVEFILP
EEPEPTEESQLASTLDNIMFITVGGRERTQKQYENMCKLAGFSKFQVACRAFSSLGVMEFY
K
>HvuOMT
MDKISAPFFSGTSPAAASVAGVDEDDRLCFQAQELMFAYNISMVLRAAIQLGLLDALSAA
GGKALTPNELVENVETSSNKAEAAAAVDRILRYLSCFNVVTCSSEAAGPDGTLVRRYTTGP
LCRWLTKDRGDGTLSPFAVFVVDPDHLFPWHHIAEAVTAGGPSAFERTQKWPYYEYMGK
NQRLGTLFDNAMAQHSVILVTKMLERFKGFDGVQRLVDVGGGTGSTLGMITSKYKHMT
GINYDLPHVIAQGLPLPGVEHVAGDMYESIPTGDAVLLQWITLMLNDDEFVKILSNCHNA
LPKDGKVIVVDGILPENPDSSLTARDAFTLDIIMFVLFKGAKQRTEKEFARLAKQAGFTGGI
KKTYIFFNFYALEFTK
>HvuF7OMT
MQDTSSTQHKSLPNNIIEMDMVTSMPLEANSNGQILQAEAELFCHSFGYLKSMALQSVVK
LRIPDVLHRYGGAASLPELLSTVPIHPNKLPYLPRLMKMLAAAGIFTAEDVPATVGDGEPT
TLYHLNAVSRLLVDDASVNGGASMSPCVLLGTVPLFLGASLKLHEWLQSEEQATTETPFM
LAHGGTLYGIGGRDSEFNTVFNKAMGASSEFVAALAVRECRDVFAGIKSLVDVAGGNGTT
ARTIAEAFPYVKCSVLDLPQVIQGISSHGTVEFVAGDMMEFVPPAEAVLLKYVLHNWSDQ
DCVKILTRCREAISHGEKAGKVIIIDTVVGSPSQQILESQVTMDLSMMMLFNGKVREEQN
WHKIFLEAGFSHYKIHNVLGMRSLIEVQP
>Msa7OMT
MASSINGRKPSEIFKAQALLYKHIYAFIDSMSLKWAVGMNIPNIIHNHGKPISLSNLVSILQV
PSSKIGN
VRRLMRYLAHNGFFEIITKEEESYALTVASELLVRGSDLCLAPMVECVLDPTLSGSYHELK
KWIYEEDLT
LFGVTLGSGFWDFLDKNPEYNTSFNDAMASDSKLINLALRDCDFVFDGLESIVDVGGGTG
TTAKIICETFPKLKCIVFDRPQVVENLSGSNNLTYVGGDMFTSIPNADAVLLKYILHNWTD
KDCLRILKKCKEAVTNDGKRGKVTIIDMVINEKKDENQVTQIKLLMDVNMACLNGKERN
EEEWKKLFIEAGFQHYKISPLTGFLSLIEIYP
>MsaCa3OMT
MGSTGETQITPTHISDEEANLFAMQLASASVLPMILKSALELDLLEIIAKAGPGAQISPIEIAS
QLPTTN
PDAPVMLDRMLRLLACYIILTCSVRTQQDGKVQRLYGLATVAKYLVKNEDGVSISALNLM
NQDKVLMESWYHLKDAVLDGGIPFNKAYGMTAFEYHGTDPRFNKVFNKGMSDHSTITM
KKILETYTGFEGLKSLVDVGGGTGAVINTIVSKYPTIKGINFDLPHVIEDAPSYPGVEHVGG
DMFVSIPKADAVFMKWICHDWSDEHCLKFLKNCYEALPDNGKVIVAECILPVAPDSSLAT
KGVVHIDVIMLAHNPGGKERTQKEFEDLAKGAGFQGFKVHCNAFNTYIMEFLKKV
>MsaI12'OMT
MGNSYITKEDNQISATSEQTEDSACLSAMVLTTNLVYPAVLNAAIDLNLFEIIAKATPPGAF
MSPSEIAS
KLPASTQHSDLPNRLDRMLRLLASYSVLTSTTRTIEDGGAERVYGLSMVGKYLVPDESRG
YLASFTTFLCYPALLQVWMNFKEAVVDEDIDLFKNVHGVTKYEFMGKDKKMNQIFNKS
MVDVCATEMKRMLEIYTGFEGISTLVDVGGGSGRNLELIISKYPLIKGINFDLPQVIENAPP
LSGIEHVGGDMFASVPQGDAMILKAVCHNWSDEKCIEFLSNCHKALSPNGKVIIVEFILPE
EPNTSEESKLVSTLDNLMFITVGGRERTEKQYEKLSKLSGFSKFQVACRAFNSLGVMEFYK

>CNtaCaOMT
MESSTKSQIPTQSEEERNCTYAMQLLSSSVLPFVLHSTIQLEVFEILAKSNDTKLSASQIVSQ
IPNCKNP
DAATMLDRMLYVLASYSLFTCSIVEDEENNGGQKRVYGLSQVGKFFVRDEDGASMGPLL
ALLQDKVFINSWFELKDAVLEGGVPFDRVHGVVHAFEYPKSDPKFNDVFNKAMINHTTV
VMKKILENYKGFENLKTLVDVGGGLGVNLKMITSKYPTIKGTNFDLPHVVQHAPSYPGV
EHVGGDMFESVPEGDAIFMKWILHDWSDSHNLKLLKNCYKALPDNGKVIVVEAILPVKP
DIDTAVVGVSQCDLIMMAQNPGGKERSEEEFRALATEAGFKGVNLICCVCNFWVMEFCK
>NtaCCoA3OMT5
MAENGIKHQEVGHKSLLQSDALYQYILETSVYPREPESMKELREVTAKHPWNLMTTSAD
EGQFLNMLLKLINAKNTMEIGVYTGYSLLATALAIPDDGKILAMDINRENYEIGLPIIEKAG
VAHKIEFREGPALPVLDQLVEDKKNHGTYDFIFVDADKDNYINYHKRIIDLVKVGGLIGYD
NTLWNGSVVAPPDAPMRKYVRYYRDFVLELNKALAVDPRIEICMLPVGDGITLCRRIT
>ObaCaOMT
MGSATNTPQINSDEEENFLFAMQLASASVLPMVLKSAIELDLLELIKKSGAGAFVSPVDLA
AQLPTTNPDAHVMLDRILRLLTSYAILECRLKTLPDGGVERLYGLAPVCKFLTKNEDGVS
MAPLTLMNQDKVLMESWYHLSDAVVDGGIPFNKAYGMTAFEYHGTDPRFNKVFNQGMS
NHSTITMKKILETYTGFDGLKTVVDVGGGTGATLNMIVSKYPSIKGINFDLPHVIEDAPSY
PGVEHVGGDMFVSVPKGDAIFMKWICHDWSDEHCVKFLKNCYDALPQNGKVILAECVL
PEAPDTGLATKNVVHIDVIMLAHNPGGKERTEKEFQGLAKAAGFKQFNKACCAYNTWIM
ELLK
>ObaChOMT
MALQNMDISLSTEQLLQAQAHVWNHMYAFANSMSLKCAIQLGIPDILHKHDHPMTLSQL
LKAIPINKEKSQSFQRLMRALVNSNFFIEENSNNQEVCYWLTPASRLLLKGAPLTVAPLVQV
VLDPTFTNPWHYMSEWFKHENHATQFEAANGCTFWEKLANKPSMGRFFDEAMSCDSRL
VAHVLTKDYKHVIDGIRTLVDVGGGNGTMAKAIVEAVPTMKCTVLDLPHVVAGLESTDK
LSYIGGDMFQSIPSADAILLKFIIHDWDDEEGLKILKRCKDAVGIGGKVIIIDVVVGVNHDV
DEVLEDQLHFDMAMMSYFNAKERTMNEWEKLISAAGFTSYKLTPAFGVRSLIEAYP
>ObaEuOMT
MALQKVDISLSTEQLLQAQVHVWNHMYAFANSMSLKCAIQLGIPDILHKHGRPMTLSQL
LQSIPINKEKTQCFQRLMRALVNSNFFIEENNSNNQEVCYWLTPASCLLLKEAPLTVTPLVQ
VVLDPTFTNPWHHMSEWFTHEKHATQFEAANGCTFWEKLANEPSKGRFFDEAMSCDSR
LIAHVFTKDYKHVIEGIRTLVDVGGGNGTMAKAIVEAMPTIKCTVIDLPHVVAGLESTDNL
NYIGGDMFQSIPSADAILLKSIIHDWDDVEGLKILKKCKDAVVMGGKVIIIDVVVGVNHDI
DEVLEDQLHFDMAMMCYFNAKERTMSEWEKLIYDAGFKSYKLTPAFGVRSLIEAYP
>Pso4'OMT1
MGSSIDAETHEVDIKDQAQLWNIIYGYADSLVLRCTVEIGIADIIKNNNGSITLSELVSKLPL
SNVNSDN
LYRLLRYLVHLNILGQQTCAAGVDRVYSLKPVGTLLLKDSERSMAPVILGLSQKDFLFVW
NFVKEGLGTGSTTAFEKAMGMDMWKYLEVNPNQSQLFDEGQAGETRLLTKTLLVDCRD
TFQGMDSLVDVGGGNGTTIKAIHEAFPHIKCTLYDLPHVIANSDDHPNILKVPGDMFMSVP
SAQVLLLKCVLHDWTDEHCVNILKKCKEAIPKETGKVIIVDVALEEESEHELTKARLILDID
MLVNTGGRERTAEDWENLLKRAGFRSHKIRPIRAIQSVI
EAFP

>Pso4'OMT2
MGSLDAKPAAATQEVSIKDQAQLWNIIYGFADSLVLRCAVEIGIADIIKNNDGAITLAQLAA
KLPITNVSSDYLYRMVRYLVHLNIIEQETCNGGVEKVYSLKPVGTLLLRDAERSMVPMILG
MTQKDFMVSWHFMKEGLGNGSTTAFEKGMGMDIWKYLEGNPDQSQLFNEGMAGETRL
LTKTLIEDCRDTFQGLDSLVDIGGGNGTTIKAIYEAFPHIKCTLYDLPHVVANSHDLPNIEK
VPGDMFKSVPSAQAILLKLILHDWTDEECVNILKKCKEAIPKETGKVIIVDVALEEESNHEL
TKTRLILDIDMLVNTGGRERTADDWENLLKRAGFRSHKIRPIRAIQSVIEAFP
>Pso6OMT
METVSKIDQQNQAKIWKQIYGFAESLVLKCAVQLEIAETLHNNVKPMSLSELASKLPVAQP
VNEDRLFRIMRYLVHMELFKIDATTQKYSLAPPAKYLLRGWEKSMVDSILCINDKDFLAP
WHHLGDGLTGNCDAFEKALGKSIWVYMSVNPEKNQLFNAAMACDTRLVTSALANECKS
IFSDGISTLVDVGGGTGTAVKAISKAFPDIKCTIYDLPHVIADSPEIPNITKISGDMFKSIPSAD
AIFMKCILHDWNDDECIQILKRCKEALPKGGKVIIVDVVIDMDSTHPYAKIRLTLDLDMML
NTGGKERTKEEWKTLFDAAGFASHKVTQISAVQSVIEAYPY
>Pso6OMT2
METVSKIDQQNQAKIWKQIYGFAESLVLKCAVQLEIAETLHNNVKPMSLSELASKLPVAQP
VNEDRLFRIMRYLVHMELFKIDATTQKYSLAPPAKYLLRGWEKSMVDSILCINDKDFLAP
WHHLGDGLTGNCDAFEKALGKSIWVYMSENPEKNQLFNAAMACDTRLVTSALANECKS
IFSDGISTLVDVGGGTGTAVKAISKAFPDIKCTIYDLPHVIADSPEIPNITKISGDMFKSIPSAD
AIFMKCILHDWNDDECIQILKRCKEALPKVGKVIIVDVVIDMDSTHPYAKIRLTLDLDMML
NTGGKERTKEEWKTLFDAAGFASHKVTQISAVQSVIEAYPY
>Pso7OMT
MDTAEERLKGQAEIWEHMFAFVDSMALKCAVELGIPDIINSHGRPVTISEIVDSLKTNTPSS
SPNIDYLT
RIMRLLVHKRLFTSELHQESNQLLYNLTRSSKWLLKDSKFNLSPLVLWETNPILLKPWQYL
GKCAQEKSSPFERAHGCEIWDLALADPKFNNFLNGAMQCSTTTIINEMLLEYKDGFSGIA
GSLVDVGGGTGSIIAEIVKAHPHIQGINFDLPHVVATAAEFPGVKHVGGDMFVDIPEADAVI
MKWILHDWSDEDCTIILKNCYRAIRKKKNGKVIIVDCVLRPDGNDLFDKMGLIFDVLMM
AHTTAGKERTEAEWKILLNNAGFPRYNVIRTPAFPCIIEAFPE
>PsoCaOMT
MGSIRNVDEDIQACRYAMELASASVPVMVLKTAIELDILEIIKKPGPGTQISVSEIVSQIQNL
KNPDAPV
MLDRMLRLLASYNILTCSLKDGGNDDKVERLYGLAPVCKFLTKNEAGCSMSALLLMNQD
KVLMESWYHLKDAILDGGIPFNKAYGVHAFEYHGKDLRFNKVFNKGMSDHTTITMNKIL
ETYKGFEGLNSIVDVGGGVGATVSMIISKYPTIQGINFDLPHVIQDAPSFPGIKHVGGDMFA
SVPNADAIFMKWILHDWSDEHSVKILKNCYDALPKNGKVIIVECIIPEVSDSSVAGHGVFH
LDNIMLAHNPGGKERSLKQFENLAKDTGFTDFQVVCSAYDTYVMEFYKN
>PsoOMT2
MEIQMGDQEQEMKNQGLVWNKICGSVDSSVLKCTIELGLLDTINSSSSKPMITLSELSSTS
PSLHSIKTQNLYRLLRYLTHMNLITINSTEATESFSLTDLAKLLLKTQERSLKDWVLGIDDE
HSVNGWHELSSYCCSPDDAPTPFVKLHGKTLWELAEEVPEVNELINNGMACDTRMVMP
AFVQGCEKVLSGIRKLVDIGGGTGAAISYVVKAFPQIKGTVLDLPHVVATAPEIPGVEMVG
GDMFESIPHADALLLKFMLHNWKDEECIKLLKRCKEAIPTDQGKVIIIEMVLDQDEDDED
LTQARLSLDLDMMLSSGGKERTKDEWRVLLESAGFSRIEFIPIYAIQSVIVAYP

>PsoOMT
MEATKSDQANQANIWKLIYGFAESLVLKCAIQLEIADTIHNHGEPMSLSELASKLPVQPVN
SDRLYRVMRYLVHMKLFNKEKTSINGEFKYSLAPPAKFLIKGWEKSMVASILAINDKDFLA
PWHHLKDGLSGDCDAFEKALGKSIWVYMSENPEKNQLFNEAMACDTRLVTSALVNDCQ
SVFKGINTLVDVGGGTGTAVKAISKAFPHIKCSIYDLPHVIADSPEIPNVVKIEGDMFKAIPS
ADAILMKCILHDWNDDECIQILKKCKEAVPQEGGKVIIVDVVLNMDLTHPYSKIRLTLDLD
MMLNTGGKERTVEEWKKLIDAAGFASFKITEISAVQSVIEAFPY
>PsoOMT3
MEVVSKIDQENQAKIWKQIFGFAESLVLKCAVQLEIAETLHNNVKPMSLSELASKLPAQPV
NEDRLYRILHFLVHMKLFNKDATTQKYSLAPPAKYLLKGWEKSMVPSILSVTDKDFTAPW
NHLGDGLTGNCNAFEKALGKGIRVYMRENPEKDQLFNEGMACDTRLFASALVNECKSIFS
DGINTLAGVGRGTGTAVKAISKAFPDIKCTIHDLPEVTSKNSKIPRDVFKSVPSADAIFMKSI
LHEWNDEECIQILKRCKEAIPKGGKVIIADVVIDMDSTHPYSKSRLAMDLAMMLHTGGKE
RTEEDWKKLIDAAGFASCKITKLSALQSVIEAYPH
>PsoN7OMT
MEVVSQIDQENQAIIWKQIYGFSESLLLKCAVQCEIAETIHNHGTPMSILELAAKLPIDQPV
NIDRLYRV
MRYLVHQKLFNKEVISTLNGGTVQVTEKYWLAPPAKYLIRGSQQSMVPSVLGIIDEDMFA
PWHILKDSLTGECNIFETALGKSISVYMSENPEMNQISNGAMAFDSGLVTSHLVNECKSVF
GDEIKTLVDVGGGTGTALRAISKAFPNIKCTLFDLPHVIADSPEIPTITKVSGDMFKSIPSAD
AIFMKNILHDWNDDECIQILKRCKDVVSAGGKLIMVEMVLDEDSFHPYSKLRLTSDIDMM
VNNGGKERTEKEWEKLFDAAGFASCKFTQMSVGFAAQSIIEVY
>PsoSOMT
MATNGEIFNTYGHNRQTATVTKITASNESSNGVCYLSETANLGKLICIPMALRAAMELNVF
QLISKFGTDAKVSASEIASKMPNAKNNPEAAMYLDRILRLLGASSILSVSTTKKSINRGGD
DVVVHEKLYGLTNSSCCLVPRQEDGVSLVEELLFTSDKVVVDSFFKLKCVVEEKDSVPFE
VAHGAKIFEYAATEPRMNQVFNDGMAVFSIVVFEAVFRFYDGFLDMKELLDVGGGIGTSV
SKIVAKYPLIRGVNFDLPHVISVAPQYPGVEHVAGDMFEEVPKGQNMLLKWVLHDWGDE
RCVKLLKNCWNSLPVGGKVLIIEFVLPNELGNNAESFNALIPDLLLMALNPGGKERTISEY
DDLGKAAGFIKTIPIPISNGLHVIEFHK
>PsoNaOMT
MEIHLESQEQEMKYQSQIWNQICGTVDTSVLRCAIQLGIFDAIHNSGKPMITLTELSSIVSSP
SSSSIEP
CNLYRLVRYLSQMDLISIGECLNEATVSLTGTSKLLLRNQEKSLIDWVLAIXCEMMVVVW
HELSSSVSTPADEPPIFQKVHGKNALELAGEFPEWNDLINNAMTSDTSVTKPALIQGCGKIL
NGVTSLIDVGGGHGATMAYIVEAFPHIKGAVIDLPHVVEAAPERPGVEFISGDIFKSISNAD
AVLLKYVLHNWEDTECVNLLKRCKEAVPADKGKVIIMDLVIDDDDNSILTQAKLSLDLTV
MNHGGGRERTKEDWRNLIEMSGFSRHEIIPISAMPSIIVAYP
>Psa6aOMT
MDFSTNGSEESELYHAQIHLYKHVYNFVSSMALKSAMELGIADAIHNHGKPMTLPELSSS
LKLHPSKVNILYRFLRLLTHNGFFAKTTVKSNEGEEETAYVLTPSSKLLVSGKSTCLSSLVK
GALHPSSLDMWGVSKKWFHEDKEQTLFECATGENYWDFLNKDSDSLSMFQDAMAADS
RLFKLAIQENKHVFEGLESLVDVAGGTGGVAKLIHEAFPHIKCTVFDQPQVVGNLTGNENL
NFVGGDMFKSVPSADAVLLKWVLHDWNDELSLKILKNSKEAISHKGKDGKVIIIDISIDEN

SDDRGLTELQLEYDVVMLTMFLGKERTKKEWEKLIYDAGFSRYKITPIC
GFKSLIEVYP
>PtaCaOMT
MDSNMNGLAKSNGCEISRDGFFESEEEELQGQAEAWKCTFAFAESLAVKCVVLLGIPDMI
AREGPRATLSLCEIVANLPTESPDAACLFRIMRFLVAKGIFPASKSARRRAFETRYGLTPASK
WLVKGRELSMAPMLLMQNDETTLAPWHHFNECVLEGGVAFQKANGAEIWSYASDHPDF
NNLFNNAMACNARIVMKAILSKYQGFHSLNSLVDVGGGTGTAVAEIVRAYPFIRGINYDLP
HVVATASSLSGVQHVGGDMFETVPTADAIFMKWIMHDWNDEDCIKILKNCRKAIPDTGK
VIIVDVVLDADQGDNTDKKRKKAVDPIVGTVFDLVMVAHSSGGKERTEKEWKRILLEGGF
SRYNIIEIPALQSVIEAFPR
>PtrCaOMT
MGSTGETQMTPTQVSDEEAHLFAMQLASASVLPMILKTAIELDLLEIMAKAGPGAFLSTSE
IASHLPTKNPDAPVMLDRILRLLASYSILTCSLKDLPDGKVERLYGLAPVCKFLTKNEDGV
SVSPLCLMNQDKVLMESWYYLKDAILDGGIPFNKAYGMTAFEYHGTDPRFNKVFNKGM
SDHSTITMKKILETYKGFEGLTSLVDVGGGTGAVVNTIVSKYPSIKGINFDLPHVIEDAPSY
PGVEHVGGDMFVSVPKADAVFMKWICHDWSDAHCLKFLKNCYDALPENGKVILVECILP
VAPDTSLATKGVVHVDVIMLAHNPGGKERTEKEFEGLAKGAGFQGFEVMCCAFNTHVIE
FRKKA
>PtrCCoA3OMT
MATNGEEQQSQAGRHQEVGHKSLLQSDALYQYILETSVYPREPECMKELREVTAKHPWN
IMTTSADEGQFLNMLLKLVNAKNTMEIGVYTGYSLLATALAIPEDGKILAMDINRENYEL
GLPVIQKAGVAHKIDFKEGPALPVLDQMIEDGKYHGSFDFIFVDADKDNYINYHKRLIELV
KVGGLIGYDNTLWNGSVVAPPDAPMRKYVRYYRDFVLELNKALAADPRIEICMLPVGDG
ITLCRRIQ
>ParOMT
MGSVRASHELLQAQAHIWNHIFSFINSLSLKCAVQLDIPDVIQKHGQPMTLSELVSALPISP
TKAHFIPRLMRILVHSGFFAKESLSGCGEQGYILTDASALLLKDNPMSARPFLLAMLSPILT
DPYQYLTTWFQNDNPTPFHVVNGMTCWDYVNQDPTLAHFFNDAMASDAQLISSLVIDDC
KEVFQGVDSLVDVGGGTGTVAKSIADAFPHMKCTVLDLPHVVADLKGSKNLEYVAGDM
FEAVPAADAIFLKWILHDWSDEECVKILERCKAAVTREGKKGKVIIVEMTVENKNTDKES
GETQLFFDMHMMVMSTGKERNEKEWAKLFSDAGFSQYKITPLFGFKSLIEVYP
>PduCaOMT
MGSTGETQMTPTQVSDEEANLFAMQLASASVLPMVLKAAIELDLLEIMAKAGPGVFLSPT
DIASQLPTKNPDAPVMLDRMLRLLASYSILTYSLRTLADGKVERLYGLGPVCKFLTKNEEG
VSIAPLCLMNQDKVLLESWYHLKDAVLEGGIPFNKAYGMTAFEYHGTDPRFNKVFNRGM
ADHSTITMKKILETYKGFEGLTSVVDVGGGTGAVLNMIVSKYPSIKGINFDLPHVIEDAPQ
YPGVEHVGGDMFVSVPKGDAIFMKWICHDWSDEHCLKFLKNCYAALPDNGKVILGECIL
PVAPDSSLATKGVVHIDVIMLAHNPGGKERTEQEFQALAKGAGFQGFNVACSAFNTYVIE
FLKKN
>PduOMT
MDLSNEMSSANLLQAQAHIWNCIFSFINPMSLKCAVQLGIPDIIKKHGNPMSLSDLISALPI
HPKKSNCVYRLMRILVHSGFFCRQKLSELDEEEGYVLTDASRLLLKDDPLSARPFLLGALD
PFMTKPWHYFSTWFQNDDPTACVTAHGTTFWDFGCLEPSLSHIFNDAMASDARLISKVVS
NEYKGVFEGLESLVDVGGGIGTMPKAIADVFPHVECIVFDLPHVVADLKGSENLKFFGGD

MFEAIPPTDAILMKWILHDWSDEECIKILQRSKEAITRKEKKGKVIIVDMVMKQKGDDQSI
ETQLFFDMLMMVLFTGKERTEKEWAKLFSDAGFSDYKITPICGLRYLIEVYP
>PpyOMT
MSSSNAVGATSHELLGAQAQLWNHIFQFINSMSLKCAVQLGIADVIHNHGQPISLSELIAGL
NVHPSKAHFVSRLMLILVHSNFFAQHHHVHHDRADVEEEEAVVLYSLTPSSRLLLKDGPFS
TTPFLLATLDPVVTTPFHLMGAWLKINGGDDPGATCTPFEMENGMPFWELGAQEPRFGNL
FNEAMEADSKLIGRVVVEECGGVFEGLKSLMDVGGGSGTMAKAIANAFPNINCTVFDQP
HVVAGLQGTTHNLGFMGGDMFEEIPPANAILLKWIMHDWNDEESVTILKKCREAISLSKN
EGGNKKIIIIDIVVGYVDNKKKMMDKKSIETQLMFDMLMMSILPGKERSKLEWEKIFFSA
GFTHYNITHTLGLRSLIEVYP
>PraCaOMT
MDSNMNGLAKSNGCEISRDGFFESEEEELQGQAEAWKCTFAFAESLAVKCVVLLGIPDMI
AREGPRATLSLGEIVAKLPTESPDAACLFRIMRFLVAKGIFRASKSAREGGAFETRYGLTPAS
KWLVKGRELSMAPMLLMQNDETTLAPWHHFNECVLEGGVAFQKANGAEIWSYASDHPD
FNNLFNNAMACNARIVMKAILSKYQGFHSLNSLVDVGGGTGTAVAEIVRAYPFIRGINYDL
PHVVATASSLSGVQHVGGDMFETVPTGDAIFMKWIMHDWNDEDCIKILKNCRKAIPDTG
KVIIVDVVLDADQGDNTDKKRKKAVDPIVGTVFDLVMVAHSSGGKERSEKEWKRILLEG
GFSRYNIIEIPALQSVIEAFPR
>RhcOrOMT
MERLNSFRHLNQKWSNGEHSNELLHAQAHIWNHIFSFINSMSLKSAIQLGIPDIINKHGYP
MTLSELTSALPIHPTKSHSVYRLMRILVHSGFFAKKKLSKTDEEGYTLTDASQLLLKDHPLS
LTPYLTAMLDPVLTNPWNYLSTWFQNDDPTPFDTAHGMTFWDYGNHQPSIAHLFNDAMA
SDARLVTSVIINDCKGVFEGLESLVDVGGGTGTLAKAIADAFPHIECTVLDLPHVVADLQG
SKNLKYTGGDMFEAVPPADTVLLKWILHDWSDEECIKILERSRVAITGKEKKGKVIIIDMM
MENQKGDEESIETQLFFDMLMMALVGGKERNEKEWAKLFTDAGFSDYKITPISGLRSLIE
VYP
>She6OMT
MEAQKENISSQAKLWNFIYGFAESLVLKCAVELDFANIIHNHGKPMTLSELASQLPVMQPV
NTNSLYRVMRYLVHINIFTKTLDENDGETKYGLAAPAKFLVKGWDNCMVGSILGITDKVF
MEPWYYIKDELAPGTGTAFELALGKDIWEYMGENPEKNKLFNAAMACDTSMIMSALISE
CKDKFNGIRTLVDVGGGTGTAARNIARAFPNIKCTVYDLPHVIADSPVYPEINRVSGDMFK
CIPNADAILMKCILHDWEDKECIEILKRCKEAVPVDGGKVIIIDVVLDGESEHPYTKVRLNS
DLDMMLNTEGKERTKEGWKKLFKAAGYRDYNITQISALQSVIEAFPY
>Tfl4'OMT
MAAFQGKDASVDIKAQAHVWKIIYGFADSLVLRCAVELGIFDIIDNNNQPIPLADLASKLPI
SNVNLDNLFRILRYLVKMELLSYAADDKYALEPIAKLVLRNEKRSMVPMVLGMTQKDFM
TPWHSMKDGLTDNGVTAFEKAMGMTIWEYLEGHPDQSQLFNEGMAGETRLLTSSLISGS
KDMFQSIDSLVDVGGGNGTTIKAISVAFPHIKCTLFDLPHVIANSYDHPNIERIGGDMFKG
MPSAQAILLKLILHDWNDEDSIKILKQCRKAVPKDGGKVIIVDVALDEDSDHELSSTRLILD
IDMLVNTGGKERTKEDWEKLVKCAGFSGCKIRHIAAIQSVIEVFP
>Tfl6OMT
MEMINKENLSSQAKLWNFIYGFADSLVLKSAVQLDLANIIHNHGSPMTLSELSLHLPSQPV
NQDALYRVLRYLVHMKLFTKSSIDGELRYGLAPPAKFLVKGWDKCMLGAILTITDKDFMA
PWHYLKEGILNDGSTSTAFEKALGTNIWDYMAEHPEKNQLFNEGMANDTRLIMSALVKE

CSSMFDGITTIVDVGGGTGTAVRNIAKAFPHIKCTVYDLPHVIADSPGYTEINSIQGDMFKY
IPNADAIMMKCILHDWDDKECIEILKRCKDAVPRDGGKVIIIDIILDVKSEHPYTKMRLTLD
LDMMLNTGGKERTEEEWKKLIHDAGYKGYKITHISAVQSVIEAYPY
>TflSOMT
MALQEGVNYLSGLGLSRLICLPMALRAAIELNVFEIIFQAGPEAQLSPAEIVAKIPTKNPNA
AIALDRIL
RMLGASSILSVTTMKDGRVYGLTEESRCLVADKNGVSVVPMLLFTSDKAVVESFYNIKDV
VLEEGVIPFDRTHGMDFFAYAGKEQSVNKSFNQAMGAGSTIAFDEVFKVYKGFHDLKELV
NVGGGIGTSLSNIIFKYPHIKGINFELPHVIADAPNYPGVEHIAGNMFEGVPNAQNILLKWV
LHDWDDERSIKILQNCWKALPEGGTVIVVEFVLPQILGNNAESFNALTPDLLMMTLNPGG
KERTTTEFDGLAKAAGFAETKFFPISQGLHVMEFHKATAGVAS
>TtuCaOMT2
MGSTQKNHNLTPEEEEEACLHAMQLASASVLPMVLKAAIELSVLEIIAKAGQGAYVAPTEI
ASQLSTSNSQAPIILDRILRLLASYKVLTCNLRTLEDGGVERLYGLAPVCKFLVKNEDGVSI
APLVLMNQDKVLMESWYHLKDAVLDGGIPFNKAYGMTAFEYHGTDPRFNKVFNRGMA
DHSTITMKKLLELYKGFEGLKSVVDVGGGTGATINMIVTKHPTIKGINFDLPHVIDDAPAY
PGVEHIGGDMFVSVPKGDAIFMKWILHDWSDEHSVKFLKNCYESIPADGKVIIVESILPVY
PETNLASNACFQLDNIMLAHNPGGKERTEKDFEALSAKAGFTGFKIVCGAFGSWVMEFC
K
>TtuCaOMT4
MGSTENNHNLTPAEEEEEEACLHAMQLASASVLPMVLKAAIELDVLEIIAKAGKGAYVAP
SEIASQLSTSNSQAPTVLDRMLRLLASYKVLTCNLRNLEDGGVERLYGLAPVCKFLVKNE
DGVSMAPLVLMNQDKVLMESWYHLKDAVLDGGIPFNKAYGMTAFEYHGTDPRFNKVFN
RGMADHSTITMKKLLELYKGFEGLKSVVDVGGGTGATVNMIVTKHPTIKGINFDLPHVID
DAPAYPGVEHIGGDMFVSVPKGDAIFMKWILHDWSDEHSVKFLKNCYESIPADGKVIIVES
VLPVFPETNLAAHTCFQLDNIMLAHNPGGKERTEKDFKALSVKAGFTGFKVVCGAFGSW
VMEFCK
>TtuCaOMT
MGSTENNHNLTPAEEEEEEAYLHAMQLASASVLPMVLKAAIELDVLEIIAKAGKGAYVAP
SEIASQLSTSNSQAPTVLDRMLRLLASYKVLTCNLRNLEDGGVERLYGLAPVCKFLVKNE
DGVSMAPLVLMNQDKVLMESWYHLKDAVLDGGIPFNKAYGMTAFEYHGTDPRFNKVFN
RGMADHSTITMKKLLELYKGFEGLKSVVDVGGGTGATVNMIVTKHPTIKGINFDLPHVID
DAPAYPGVEHIGGDMFVSVPKGDAIFMKWILHDWSDEHSVKFLKNCYESIPADGKVIIVES
VLPVFPETNLAAHTCFQLDNIMLAHNPGGKERTEKDFKALSVKAGFTGFKVVCGAFGSW
VMEFCK
>TtuCaOMT3
MGSTQKNHNLTPEEEEEACLHAMQLASASVLPMVLKAAIELDVLEIIAKAGQGAYVAPTE
IASQLSTSNSQAPIILDRVLRLLASYKVLTCNLRTLEDGGVERLYGLAPVCKFLVKNEDGVS
IAPLVLMNQDKVLMESWYHLKDAVLDGGIPFNKAYGMTAFEYHGTDPRFNKVFNRGMA
DHSTITMKKLLELYKGFEGLKSVVDVGGGTGATINMIVTKHPTIKGINFYLPHVIDDAPAY
PGVEHIGGDMFVSVPKGDAIFMKWILHDWSDEHSVKFLKNCYESIPADGKVIIVECILPVY
PETNLASNACFQLDNIMLAHNPGGKERTEKDFEALSAKAGFTGFKIIC
GAFGSWVMEFCK
>TtuCaOMT5

MGSTQNNHNLTPEEEEEACLHAMQLASASVLPMVLKAAIELDVLEIIAKAGQGAYVAPSE
IASQLSTSNSQAPIILDRILRLLASYKVLTCNLRTLEDGGVERLYGLAPVCKFLVKNEDGVS
MAPLVLMNQDKVLMESWYHLKDAVLDGGIPFNKAYGMTAFEYHGTDPRFNKVFNRGM
SDHSTITMKKLLELYKGFEGLKSVVDVGGGTGATVNMIVTKHPTIKGINFDLPHVIEDAPA
YPGVEHIGGDMFVCVPKGDAIFMKWILHDWSDEHSVKFLKNCYESIPADGKVIDVESILP
VCPETNLAANACFQLDNIMLAHNPGGKERTEKDFEALSVKAGFTGFKVVCGAFGSWVM
EFCK
>TaeOMT
MALTGDYKLISTDDMLQGHAELCIHAYGFVKSMALKCAIELGIPGAIHGHGGGATLGELA
TIIALPPSRL
PRLRRLMRVLTVSGVFSVQNQQPDDPAGCAAVVYGLTAASRLLVGDGESIVGAAPPRSLM
VDPNLTAPFSGMSAWFMDDEQPRSFFEMHHGEDMWEMAARDAALSRTIGDGMTDDSRF
VVEVLLREGRARDVFSGVRSMVDVGGGTGTIAKAIAAAFPHVECSVLDLPHVVAEAPAG
GEVRFIEGDMFEHIPPADAVLLKSVMHDWRDDECVKILRRCKEAIPSREAGGKVIIINMVV
GSEKSKGNSTKKGGGTSIVRSSSSWFLREVNEKSMSGRRSFLEAGFSGYSIIPMLGIRSIIEV
YP
>VviCaCoAOMT
MATNQEAGRHQEVGHKSLLQSDALYQYILETSVYPREPESMKELRELTAQHPWNIMTTSA
DEGQFLNMLLKLINAKNTMEIGVYTGYSLLATALALPDDGKILAMDINKENYELGLPVIQ
KAGVAHKIDFKEGPALPVLDQMIEDGKYHGSFDFIFVDADKDNYLNYHKRLIDLVKVGGI
IGYDNTLWNGSVVAPPDAPLRKYVRYYRDFVLELNKALAADPRIEICMLPVGDGITLCRR
LS
>ZmaOMT
MGSTAGDVAAVVDEEACMYAMQLASSSILPMTLKNAIELGLLEVLQKEAGGGKAALAPE
EVVARMPAAPSDPAAAAAMVDRMLRLLASYDVVRCQMEDRDGRYERRYSAAPVCKWL
TPNEDGVSMAALALMNQDKVLMESWYYLKDAVLDGGIPFNKAYGMTAFEYHGTDARF
NRVFNEGMKNHSVIITKKLLDFYTGFEGVSTLVDVGGGVGATLHAITSRHPHISGVNFDLP
HVISEAPPFPGVRHVGGDMFASVPAGDAILMKWILHDWSDAHCATLLKNCYDALPENGK
VIVVECVLPVNTEATPKAQGVFHVDMIMLAHNPGGKERYEREFRELAKGAGFSGFKATYI
YANAWAIEFIK
>ZelCaCoA3OMT
MATPTGETQPAKHQEVGHKSLLQSDALYQYILETSVYPREPQPMKELRRITAKHPWNLMT
TSADEGQFLNLLLKLINAKNTMEIGVYTGYSLLSTALALPEDGKILALDINRENYEIGLPIIQ
KAGVAHKIDFREGPALPLLDQMLQDEKCHGSFDFIFVDADKDNYLNYHKRLIDLVKFGGV
IGYDNTLWNGSLVAPADAPLRKYVRYYRDFVLELNKALAVDPRVEICQLPVGDGITLCRRI
S
>ZelCa3OMT
MGSNQDDQAFLFAMQLASASVLPMVLKTAIELDLLETIAKAGPHGSVSSSELVAQLPKVN
NPEAPVMIDRICSLLASYSVLTCTLKETADGCAERFYGLAPVCKFLIKNDAGVSLAPLLLM
NQDKVLMESWYYLKDPVLDGGIPFNKAYGMSAFEYHGKDQRFNKVFNSGMFNHSTMT
MKKIVELYNGFSGLKTLVDVGGGTGASLNMITSKHKSLKGINFDLPHVIADATTYQGIEHV
GGDMFESVPKGDAIFMKWILHDWSDAHCLQVLKNCYKSLPENGKVIVAECILPEAPDTTP
ATQNVIHIDVIMLAHNPGGKERTEKEFEALAKGAGFKGFNKAACALNTWVMEFCK

P450

>CyNMCH

MEILSIAILSLVISTLIYYIFFSDSKALKGLPPGPKPWPIVGNLLQLGEKPHSQFAEMAQTYG
DLFTLKMGTQTVVVASTPSSAIEILKTHDRLLSARYVFQSFRVPNHVENSMVWSDCNEVW
KMLRKVCRTELFTPKMIESQAHVRESKAMEMVKFLKGKQGGEVKIAEVVFGTLVNIFGN
LIFSQDVFELGDPSGGSAEMKEHLWRMLELGNSTNPADYFPILGRFDLFGQRKDVAECLR
LIYDVWGVMLKERKVKKAAQGKKGNDNNDFVDVLLDSGLDDFQINALLMELFGAGTET
SSSTIEWAIAELTKNPHITAKIRAELESVVGQSPIKESDIPNLPYLQACTKETLRLHPPTPLLL
PRRAIETCQVMNYTIPKDCQIMVNAWAIGRDPKIWKDALKFSPERFLNSSVDFKGNDFELI
PFGAGRRICPGVPLATQFISLIVATLVQNFEWNLPNGMDPKDLVMDEKFGLTLQKEPPLYIV
PKSRV

>CyCYP51

MDLEENKKLLNIGILIVATLLIAKLVSVVLFPSSSKKRRRLPPTVGAWPLVGGLIRFMKGPIV
MLREEYPKLGSVFTLNLLLNKNITFFIGPEVSSHFFKASESDLSQQEVYQFNVPTFGPGVVF
DVDYSVRQEQFRFFTEALRVNKLKAYVDQMVTEAEDYFSKWGESGEVDLKYELEHVIIL
TASRCLLGREVRDKLFDDVSALFHDLDNGMLPISVIFPYLPIPAHRRRDQARKKLSQIFANII
GSRKETGKSENDMLQCFIESKYKDGRPTTESEVTGLLIAALFAGQHTSSITSTWTGAYLFQ
NKEFLSAVLDEQKSLVKKHGDKIDHDILSEMDVLYRCIKEALRLHPPLILLLRSSHTEFSVK
TPEGKEYDIPKGHIVATSPAFANRLPHVFTNPDTYDPNRFAVGREEDKAAGAFSYISFGGGR
HGCLGEPFAYLQIKAIWSHLLRNFEFELISPFPEIDWSAMVVGVKGKVMVRYKRRQLCVE

>PsNMCH1

MEIVTVSLVAVVITTFLYLIFRDSSPKGLPPGPKPWPIVGNLLQLGEKPHSQFAQLAETYGD
LFSLKLGSETVVVASTPLAASEILKTHDRVLSGRYVFQSFRVKEHVENSIVWSECNETWKK
LRKVCRTELFTQKMIESQAEVRESKAMEMVEYLKKNVGNEVKIAEVVFGTLVNIFGNLIF
SQNIFKLGDESSGSVEMKEHLWRMLELGNSTNPADYFPFLGKFDLFGQRKDVADCLQGIY
SVWGAMLKERKIAKQHNNSKKNDFVEILLDSGLDDQQINALLMEIFGAGTETSASTIEWA
LSELTKNPQVTANMRLELLSVVGKRPVKESDIPNMPYLQAFVKETLRLHPATPLLLPRRAL
ETCKVLNYTIPKECQIMVNAWGIGRDPKRWTDPLKFSPERFLNSSIDFKGNDFELIPFGAG
RRICPGVPLATQFISLIVSSLVQNFDWGLPKGMDPSQLIMEEKFGLTLQKEPPLYIVPKTRD

>PsNMCH2

MEIVTVSLVAVAITTFLYLIFRDSSPKGLPPGPKPWPIVGNLLQLGEKPHSQFAQLAETYGDL
FSLKLGSETVVIASTPLAASEILKTHDRVLSGRYVFQSFRVKEHVENSIVWSECNDTWKKL
RKVCRTELFTQKMIESQADIRESKAMEMVKYLKKNEGNEVKIVEVVFGTLVNIFGNLIFSQ
NIFKLGDESSGSVEMKEHLWRMLELGNSTNPADYFPFLGRFDLFGQRKDVADCLQGIYSV
WGAMLKERKIPKLHNNSKKNDFVEILLDSGLDDQQINALLMEIFGAGTETSASTIEWALSE
LTKNPDVTANMRSELLSVVGKRPVKESDIPNMPYLQAFVKETLRLHPATPLLLPRRALETC
KVLNYIIPKECQIMVNAWGIGRDPKRWTDPLKFSPERFLNSSIDFKGNDFELIPFGAGRRIC
PGVPLATQFISLIVPTLVQNFDWGLPKGMDPSQLIMEEKFGLTLQKEPPLYIVPKTRD

>PsNMCH5

MEIVTVALIAIVFTTFLYLIVRESSPKGLPPGPKPWPIVGNLLQLGEKPHSQFAQLAETYGDL
FTLKLGTQTVVVASTPLAASEVLKTHDRTLCGRYVFQSFRVKKHVENSIVWNECNETWK
KLRKVCRTQLFTQKMIENQAEVREIKTMEMVEYLKKNEGIEVKIVEVIFGTLVNMFGNLIF
SQDIFKLGDESSGSLEMKQHIWRMLELGNSANPGDYVPLLGSLDLFGQRKDVADCLQGV
YGVWGAMLKERKIAKQQINGDTKNDFVDVLLDSGLDDQQINSLLLDMFSAGTETTASTIE

WALTELTKNPQVTVNIRSELLSVVGKRTVKESDIPNLPYLQAFVKETLRLHPPTPLLIPRRA
LETCHVLNYTIPKECQIMVNAWGIGRDPKTWTDPLKFSPDRFLNSSIDFKGNDFELIPFGA
GRRICPGVPLATQFISLIVLAVLQNFDWELPKGMDRSQLIMEEKFGLTLQKEPPLYIVLKTR
D
>EcNMCH3
MEVVTVALVAVLISSILYLIFGGTHHKNLPPGPKPWPIVGNLLQLSDRPHSQFAELAKIYGD
LFTLRLGSETVVVASTPAAAAEIIKTHDRIFSGRYVFQSFRVKNNIENSIVWAQCNDSWKNL
RKICRTEIFTQKMIESQAHVRERKCLEMVEYLKGKQGEEVKIVEVIFGTLVNIFGNLIFSQN
VFKLGDESSGSTEMKEHLWKMLELGNSTNPADYFPILGRFDLFGHRKEVLDCLQGIYNV
WGAMLNERKSAMKLAGYERKNDFVDICLDAGLNDDQINSLFMVSSHDLFGAGTETSAS
TTEWAITELTRNPQVTAKIRSELQTVVGERPVKESDFPNLPYLQATVKETLRLHPPTPFLLP
RLALDTCQVLNYTIPKNCQVMVNAWAIGRDPKNWTDPLTFSPERFLESKVDFKGTDFELIP
FGGGRRICPGIPLANQFISLLIATMVHNFEWVLPNGMDPSRLIMEEKFGLTLQKEPPLSIVP
KSIA
>EcNMCH4
MEVVTVALIAVIISSILYLLFGSSGHKNLPPGPKPWPIVGNLLQLGEKPHAQFAELAQTYGD
IFTLKMGTETVVVASTSSAASEILKTHDRILSARYVFQSFRVKGHVENSIVWSDCTETWKN
LRKVCRTELFTQKMIESQAHVREKKCEEMVEYLMKKQGEEVKIVEVIFGTLVNIFGNLIFS
QNIFELGDPNSGSSEFKEYLWRMLELGNSTNPADYFPMLGKFDLFGQRKEVAECLKGIYAI
WGAMLQERKLAKKVDGYQSKNDFVDVCLDSGLNDYQINALLMELFGAGTETSASTIEW
AMTELTKNPKITAKLRSELQTVVGERSVKESDFPNLPYLEATVKETLRLHPPTPLLLPRRAL
ETCTILNYTIPKDCQIMVNAWGIGRDPKTWIDPLTFSPERFLNSSVDFRGNDFSLIPFGAGR
RICPGLPIANQFIALLVATFVQNLDWCLPNGMSVDHLIVEEKFGLTLQKEPPLFIVPKSRVGS
>TfNMCH
MEVLSAAMVSLFFIFLFIFFILCDSRNKDLPPGPRPSPIVGNLLQLGEKPHAEFAKLAEKYG
ELFTLKLGSQTVVVASSPAAAAEILKTRDKILSGRYVFQSFRVYEHVLNSIVWSECNENWK
LLRKVCRTELFSPKMIESQAYIREAKALDMVRFLRKKENQEVKIVEVVFNTLVNIFGNLIFS
KDVFDLEDPTGGSAELKEHLWKLLDMGNSTNPADYFPIMGKFDLFGQRREVAKVLKQIY
DVWGVMLKERRSITGHRENDFANVLLNAGLDDQKINALLMELFGAGTETTASTIEWAITE
LTNNLRVISKLRAELINVVGHKTVKESNIPHLPYLQAIVKETLRLHPPTPLLLPRRALETCK
VMNYTIPKECQIMVNAWAIGRDPKTWDDPLTFKPERFMNSTVDYKGNDFELIPFGGGRRI
CPGLPLASQFLSLIVATLVQNFDWSFPQGMTPNEVPMDEKFGLPLQKDPPLLIVLKARTSM
KLQD
>NtNMCH1
MGKMPHITLTNFAKTYGPLMSLKLGTQCLVVGSSPSAAIEILKTHDRILSGRHVPNAFPFK
KSELEKSSIGWTSECNNGWRYLRTLCRTELFSGKVLESQACLREKKVKELIEFLRSKEGQL
VNLGELIFATVLNMLSNVMISQDIVKLEKETEDGGMKSLIREAMEVGSTPNISDFYPILCKL
DLQGLRRKSMVLMSKIRSKTWERIVEERRKSKVSGSSSQHDFLETLLNNGFTNDAIHQLF
MELFTAGTDTTTSTIEWAIAELIKNEESMEKVQAELERELRESYFPKESKLLQMSYIQACV
KETLRLHPPGPFLLPHRAIETCQVMSYTIPKDAQVLVNVWAIGRDSLIWKEPEMFRPQRFL
SSNMDFKGNDFEFLPFGSGRRICPGLPMAAKSVPLVLASLVHFFDWELPNGKCSIELDMNE
KFGVTLQKREPLLLIPKARK
>NtNMCH2
MAQSYEILFLLPLFLLPLIFFLLKLCKSSKIPQLPPGPKPWPILGNIHMGKMPHITLTNFAKT

YGPLMSLKLGTQCLVVGSSPSVAIEILKTHDRILSGRHVPNAFPFKKSELEKSSIGWTSECN
NGWRYLRTLCRTELFSGKVLESQACLREKKVKELIEFLRSKEGQVVNLGELIFATVLNMLS
NVMISQDIVKLEKETEDGGMKSLIREAMEVGSTPNISDFYPILCKLDLQGLRRKSMVLMS
KIRSKTWERIVEERRKSKESGSSSQHDFLETLLNNGFTNDAIHQLFMELFTAGTDTTTSTIE
WAIAELIKNEESMEKVRAELERELRESYFPKESKLLQMSYIQACVKETLRLHPPGPFLLPH
RAIETCQVMSYTIPKDAQVLVNVWAIGRDSLIWKEPEMFRPQRFLSSNMDFKGNDFEFLPF
GSGRRICPGLPMAAKSVPLVLASLVHFFDWELPNGKCSIELDMNEKFGVTLQKREPLLLIP
KATK
>CaNMCH1
MAQPFEILCLVPLFLLPLIIFIFRVFKSSKITQLPPGPTPWPVIGNIFHMGKMPHITLTNFAKA
YGPIISLKFGTKWLVVGSSPSVAIEILKTHDRILSARHVPNAVPSEGSDLEKTSLGWNSGCSS
RWRYLRTLCKTELFSGKVLESQACLREKKVKELVEFLRSKEGQVVNIGELVIATALNMLSN
VLISKDMANFDNETEDGDIRNLVREIVEAVSAPNISDFYQILGKLDLQGLRKKSIRLVIKFCS
KWEPILEERRDSRENGSSIQEELEIELSKSDYLKESQLLQMSYVQACVKETLRLHPPAPLLL
PHRAIETCKIMSYTIPKDTQILVNVWAIARDPSMWEEPEVFRPQRFLSSDMDFKGNDFEFLT
FGAGRRICPGLPMAAIEVPLVLASLVHFFDWELPHGKCPAELDMTEKFGVTLQKKEPLLLI
PKARK
>CaNMCH2
MAQPFEILCLVPLFLLPLIIFIFRVFKSSKITQLPPGPTPWPVIGNIFHMGKMPHITLTNFAKA
YGPIISLKFGTKWLVVGSSPSVAIEILKTHDRILSARHVPNAVPSEGSDLEKTSLGWNSGCSS
RWRYLRTLCKTELFSGKVLESQACLREKKVKELVEFLRSKEGQVVNIGELVFATALNMLS
NVLISKDMANFDNETEDGDIRNLVREIVEAVSAPNISDFYQILGKLDLQGLRKKYINVAKQ
FRSKWEPILEERRNSRENGSSSQQDFLETLLDNDFTNDCIHQLFMELLSAGSDTITSTIEWA
MAELIKNVEPMKKVQEELEIELSKSDYLKESQLLQMSYVQACVKETLRLHPPAPLLLPHR
AIESCQVMSYTIPKDAQIMVNVWAITRDPLIWEEPEMFRPQRFLRSDMDFKGNDFEFLPFG
AGRRICPGLPMAAIKIPLVLASLVHFFDWELPHGKCPAELDMTEKIGVTLQKKEPLLLIPKA
RK
>CaNMCH3
MLSNVLISKDMINLDKETEDGGIKSLVRGIVEVASAPNISDFYPIFGKLDLQGLRKKSIRLVT
KFCSKWEPILEERRDSRENGSSSQQDFLETLLDNGFTNDRIHQLFMELLSAGSDTSTSTIEW
AMAELIKDVESMKKVQEELEIESESDYLKESQVLNMSFVQACVKETLRLHPPVPFLLPHR
AIETCQIMSYTIPKDAQIIVNVWAITRDPSIWEEPEMFRPQRFLSSDMDFKGNDFEFLPFGA
GRRICPGLPMAAIKVLLVVASLVHFFDWELPHGKCAAELDMTEKFGVALQKKEPLLLIPK
ARK
>SlNMCH1
MEYYTLPASFLIFIPFFLLIFKQLVPKSTNLPPGPRPWHLLGNLLQIGKTPHVSLAKFAQIHG
PLISVKLGTQLVVVASSPASAAEILKSQDRLLSVRSVPKVATYELSVIDQHSIVFSSDLSNQW
KFLRAFCRTHLFSPKAVESQAALREKKVSEMMNFLRSRKGETVKISEILFGTILNTLGNLFF
SKDLCDLDYETNTSGIKHVIRKFVELGAMPNISEFYPLFDALDLQGLRKQTEIYQNRLVNI
WSEIVKEKRQAISRSSSDFLDAMIDNGFSDLQVDILLMELISAGSDTTTSTIEWAMAELLRN
KGAMRKLKAELASKLGENDIITESNVSELPYLAACVKETLRIHPPTPFLIPRRAPETCKIMD
YTIPKNSKLFVNAWAIGRDSNTWEDALSFRPERFLNSNVDFRGQDFEFIPFGAGRRICPGL
GFARQEIQLILASLIHYFEWSLPNGEDPMQIDMKEKFGVTLQKEKSLLAVPK
>SlNMCH2

MKYLLNILTCVELMEYYMLPPSFLIFIPLLFLIVKQFIPKSGNLPPGPRSWPLLGNILQIGKN
PHISLAKFAQIHGPLISVKLGTQLVVVASSPASAAEILKTQDRLLSARSPPSVAPYELSVINQ
HSIVFSSDLSNHWKLLRAFCRTHLFSPKAVESQTALREKKVSEMIHFLKSKKGETVMISEIL
FGTILNTFGNLFFSKDLCDLDHETKTSETKRVFRKFIELGAMPNISEFYPLLEALDLQGLSK
QTKMYQNQLINIWSQIVKEKRQAVNRRSSDFLDVMIDSGFSDLQINILLTELISAGSDTTTS
TIEWAMAELLRNKGAMHKLQAELTSKIGENDIITESNISDLPYLAACVKETLRLHSPTPFLI
PRRAPETCKIMDYTIPENSKLFVNAWAIGRDSNTWEDALSFRPERFLNSNVDFRGQDFEFIP
FGAGRRICPGLGFARQEIQLILASLIHYFEWSLPNGEDPMQLDMEDKFGVTIQKEKPLLIVP
M
>LsNMCH
MTFPMVIILLLLLLPPLLALIIAVRITTKAVDVQQGNQKLPPGPYSWPIIGNLPHMLKGPHIY
LTQLARSYGPLCSVKLGTHLFVIGSSPMAATEIMRAHDKLPTYRWVPKAGQDGLQDYSLI
WATQCTDHWKLLRSLCRTELFSAKGLDLQSSLREKNVDKMVGFLKSKQGSLVNVRELVF
ASTVNILGNICFSKDFIDLDDDHKERRGLKRALYRLMKLGTTPNIADFYPRFEDPQGLKRK
TSEAMNEAFGAWEHIIKERRATREAQKDTDSSDEQDFLDTMLGSGFSDLQINQLTVELFSG
GTHSTASTIEWALAELIKNKEAMFALREELKREIGFTNYIKESQVSHLPYLHACVKETLRL
HPPAPLLHPQAILEGCEIMKYTVPKNSQLIINVWAMGRDPNLWEDPLTFKPERFCDSNVDF
KGQDFKFLPFGVGKRMCPGYPYAIKQIHLMLASLVQNFDWFLPNNIEDLSKLDMSENFGII
SQRKKPLMVIPKCKC
>VrNMCH1
MVAMLAEGTSFFDVFLPFLLLLPPLVFLILKLLKDSSSLKSPPLPPGPSPWPILGNLLHLGN
MPHISLARFSQSYGPLISLRLGSQILVVASTSSAAMEILKTHDRVLSGRYVPHAVPAKNSEIN
RMSLGWAVECNDAWKNLRTVCRAELFSTKVMESQAWVGEKKVMEMVRFVSTKEGEVM
KVGEVVFATVLNTLTTVLMSRDFISFEDDNKDGGMKGLVRKMMMAIASPNLDDFYLIFSG
LDLQGQNKKTKELIARICSMWESVIRERREGASDDPSKQDFLNMLIRSGYSDDQINQLFM
ELLTAGADTSSSTLEWAMAELIKSPESMKKVQEELAREISDNLLKASDLPHLPYLQACVKE
TLRLHPSAPLLLPRRASVSCEVMNYTIPKDSQIWVNAWAIGRDPMNWEDPLVFKPERFLN
SAVDFKGNNLEFIPFGAGRRICPGLPMAARLLSLILASLTHFFDWSLPNGTTPDELDMNDK
FGISLQKEQPLLIIPKVRK
>VrNMCH2
MVAMLAEGTSFFDVLLPFLLLLPPLVFLILKLLKDSSSLKSPPLPPGPSPWPILGNLLHLGN
MPHISLARFSQSYGPLISLRLGSQILVVASTSSAAMEILKTHDRVLSGRYVPHAVPAKNSEIN
RMSLGWAVECNDAWKNLRTVCRAELFSTKVMESQAWVGEKKVMEMVRFVSTKEGEVM
KVGEVVFATVLNTLTTVLMSRDFISFEDDNKDGGMKGLVRKMMMAIASPNLDDFYLIFSG
LDLQGQNKKTKELIARICSMWESVIRERREGASDDPSKQDFLNMLIRSGYSDDQINQLFM
ELLTAGADTSSSTLEWAMAELIKSPESMKKVHEELAREISDNLLKASDLPHLPYLQACVKE
TLRLHPSAPLLLPRRASVSCEVMNYTIPKDSQIWVNAWAIGRDPMNWEDPLVFKPERFLN
SAVDFKGNNLEFIPFGAGRRICPGLPMAARLLPLILASLTHFFDWSLPNGTTPDELDMNDK
FGISLQKEQPLLIIPKVRK
>QlNMCH1
MDQKAIVREIDYLFFFSLLLLLATLSYLYYYKHKLSKNPPLPPGPFAWPIVGNIFQMGRDLH
SILANLARTHGPLMSLRLGTQLNIIASSPAAASEILKTHDRILSGRYVTHASPIINPKLNFSIG
GAPECNDHWRSLRTICKADLFSAKVLESQKHIREKKVRELVSFLGSKEGEIVNLGGTVYIT
VTNILSNVIFSMDFLDFDGNGMGKELRKFIDEMAELMITPDLSNLYPILGALDFQGIQKKR

DKLIEKFIAIWEDIIKKRQKQESAILVHGDVLDTLIKDGHTNDQINQLIMELLMAGTVSTSI
VIEWAMAELMKNQNVMDKLHDELERGIGTDIVKESHLAHLPYLEACVKETLRLHPLGPL
LLPHRALQTCQVMGYTVPKNSQVLVNVWAIGRDPMIWNDPLSFKPERFLDSCLDIKGNNF
DYMPFGAGRKICPGQPLAIKQVRFILALLLHSFDWFLPGNMNSTELDMNDHFSLTLRKKQ
PLQLIPKGRK
>QlNMCH2
MDQKDIPLWKTDMIFFLLLFPIPLFFRYIKHVFSKNPPLPPGPFSWPIIGNIHQMGRELHSILA
NLARVHGPLMSLRLGTQTIIVASSPAAAREILKTHDKTLSGRPLSHATPVINPTLNNFSIGFAI
ECNDHWRRLRAICKAELFSAKALESKKMSREKKVMELIGFLGSKEGKVVNLGEVVYITV
TDILSNALFSVDFLDFEGQGIGKELRKLLTEDAELGITVNLSDLYPILSGLDFQGIRKKSKEI
LGKLITIWEDIIKERRKQESVISDQSDFLDALIKDGYTNDQINQLIVELFIAGIDTSIIAIEWG
VANLMKNQDAMHKLRDELDREIGTDNVKESHLAHLPYLQACVNETLRLQPPGPLLLPHR
ALQTCQVMGYTVPRDSQILVNMWAIGRDPMIWNDSLSFKPERFLDSSLDSKGNNFEYIPFS
AGRRICPGQSLATMQVPFILASLVHSFDWFLPGNMNSTELDMNEKFTITLRKKQPL
QLIPRGRK
>ZjNMCH1
MDQTLFTKMVNYISLPSLPFHLLLLLLLPVIFFIWKQFTSQSVNTPPGPKPWPIVGNLPQMS
KRGHISMTHFAKIYGPLISLRLGTRLHIIASSPSSAIEILKNHDRLLSARYIPEKLTGSKPKDA
ELSPASLMWAPSCTGHWKLLRTMCRTELFSPKAIESQTSLRASKLAEMVDFVRGREGQAV
KIKDVVFTVVFNTLSNLFFSKDLTSFEEDDYSGCKGHFRKLGEVASSLNLAEFFPILAGLDL
QGISKKTQMHAKLLTAEWEDIVKERKENRGKNSTSQRDFLDAMIENEFADHQINQLVMEL
FSAGTDTTTITVEWAMAELLKNREAMERVLEELRSVVGDQNSISESNLSQLPYLEACFKET
LRLHAPVPFLLPHCAPETCEVMNYTIPKNARILVNVWAIGRDSAFWEDPLLFKPERFIGSEL
DFKGHNYEYLPFGAGRRICPGLPMGIRQGQLILAYLLHCFDWSFPNGEDASAIDMTEKLSL
TLQMVKPLQLIPKWKL
>ZjNMCH2
MELMKFMSQIKHSEQINTLYLLLLLIPSFLFFILKRFKTSSKFKSSPLPPGPSPWPILGNILQIG
ERPHLDFTSFAKTYGPLISLRFGNQLVIIGSSPVAATEILKAQDRTLSGRYVPHVAPAKSLEV
NNLSLGWTLECNEYWRNLRTICRGELFSNTAISSFACIREKKVMEMLNLLRKMQGKVVKI
RDVTFAAVFNIVSNILVSRDLINLDDESLSGETGKLVRDIMEVASAPNISDFYPILSPFDLQG
LRKKSNELCDRSSKMWEAIIKERKETMKNGDASTQKDFLDALMNNGCPDEQINMLLLEL
LSAGTDTTSSAVEWILVELLRNPVCMKKVKEELAREIKQEKSIKDSHLTNMPYLQACMNE
AIRLHPPSPLLLPHRATESCQIMGYTIPKDAMVLVNFWAIGRDPTHWEDPLVFKPERFLNSS
LDFKGNDFQYIPFGAGRRICPGLPMAAKHVSLLAASLIHFFDWSLPRDMDPNNLDMSEKF
AISMVKDESLLVVPKIKI
>RcNMCH1
MDTAFSGVLGILSLSFLLLLPLIFIILNHISSPSSERRPLPPGPRQWPIVGNILQVGKKPHVSL
AYFAKLHGPLISLRLGAQIVVVASSPIAAAEILKTHDRLLSARFISAANPYGDHVLDRVALV
WNPSCVFTTVFNTISNLIFSKDMLSFGDQGNAGGLKTLITKLMQLATAPNIADFYPVLARL
DPQNMRRKMKIGFEKVLNAWQIYIKERRENHVNDAPETDFLDVFLSTGFDDDQINWLVL
ELFAAGVDTTTTTVEWAMAELLKSRATLVKVQQELDREVDNKSIEESHVLQLQYLNACIK
ETFRLHPPAPFLIPRRALNTCEVLNYTIPKNSQVVVNLWAIGRDSSSWEDPLSFKPERFLNS
NIDFKGHHFQLLPFGSGRRTCPGLPMATRQLPLILAYLIRCFEWSLPNDQDPAMLDMNDKF
GITLVKDSPLLLVPKRKL

>RcNMCH2
MEFFYSLNKRSNLNIPIHLRPMDQTAKLLNQFASSFCLLLLLLLPLIFIILKHIVTSLSSKRPR
LPPGPRPWPIIGNLHQIGKKLHISVTQFAKVHGPLISLRLGSRVVVVGSSPLAATEILKTHDR
LLSARTIPKVLPYKIHFVDRLAIVWASSCNERWKFLRALCRTELFSANAIESQAALREKKMI
EMVEYLSSEQGQVVNIGEVVFTSVFNTISNLIFSKDLLSFEDQERGSGLKNATWRLMELTV
APNIADFYPVFAGLDPQGLQKKMLKYMKEMFSAWEIHIKERRETHVHGAPTNDFLDVFL
ANDFDDDQINWLTFELFAAGSDTTTTTVEWAMAELLKNKQFMKKACEELDREIKRNSIKE
SDIPQLHFLNACVKETMRFHPPVPFLVHRALETCEVMNYTIPKDAQVLVNIWAIGRDPSA
WEDPLSFDPNRFLGSNIDFKGHDFELIPFGSGRRNCVGQSMATRQLHLILGSLMHFFDWSL
PNGEDSVNLDLSEKFGLTLQKEQPLLLVPKRKL
>PtNMCH1
MLVQRFSIPKLIEDRIIDNEGSLDLQGLSRKTNECGSRIQELWRSIIKERREGRNDTGDDDN
SSKRKDFLDVLLDGEFSDEQISSFFVELLAAVSDSSSSTIEWAMAELMRNPQAMKQLREEL
AGETPEDLITESSLAKFPYLHLCVKETLRLHPPAPLLIPHRATEDCQVLDCTIPKDTQVLVN
VWAIARDPASWEDPLCFKPERFLNSDLDYKGNHFEFLPFGSGRRICAGLPMAVKKVQLAL
ANLIHGFDWSLPNNMLPDELDMAEKYGITLMKEQPLKLIPKLRK
>PtNMCH2
MVSISVLANSYPSFPMLFLLAILLLLSFVLKHKSSKVPAIPPGPKSWPIIGNVLQMGNKPHIS
LTKLAQVYGPLMSLRLGTQLVVVGSSREAASEILKTHDRELSGRCVPHASFAKDPKLNED
SIAWTFECTDRWRFFRSLMRNELFSSKVVDGQSRTRETKAREMIDFLKKKEGEGVKIRDIV
FVYTFNALANIYLSKDLVDYDQIGECQRVCGLVREMMELHTTLNISDLYPILGSLDLQGVS
RKCNECESRIQELWGSVIKERREGRNDTGDDDDNSSKRKDFLDVLVDGEFSDEQISLFFVE
LLAAVSDSTSSTVDWAMAELMRNPQAMKQLREELAGETPEDLITESSLAKFPYLHFCVKE
TLRLHPPAPFLIPHRATEDCQVLDCTIPKDTQVLVNVWAIARDPASWEDPLCFKPERFLNSD
LDYKGNHFEFLPFGSGRRICAGLPMAVKKVQLALANLIHGFDWSLPNNMLPDELDMAEK
YGITLMKEQPLKLIPKLRK
>CsNMCH1
MLTPTLYFKEMLIPSFSILLPSLLLLLPPIFIILKRIKSPSSKKLPLPPGPNPWPILGNLLQIGQN
AHISITQFANIYGPLISFKLGAQRLVVASSPAAATAVLKTQDHLLSARYIFQITPDRALHDQH
SLVFSSECGDVWKKLRSICKVNLFTAKAIESQAILREKKMKELVEFVESKQGSVVEIKDFV
FTSAFNILSNLIFSMDLLDFVGDGFNGIKGPFKKMMDLGLTPNLADFYPLLRRFDLQGLIR
KTVIYKNEVNSVWGILIKERREIRCEKGSASNDFLDVLIQSGFSDQQIDALIIELLFAATDTT
TTTVEWAMSEILKDKDILNKAREEIKKVAKESPAIDESNISKLQYLWSCVKETLRLHPPVPF
LLPRLAPMDCEVMGYSIPKDSMIFVNVWGIGRDPSIWEDSQTFNPERFDVGCGNNVDFKG
YDYRYLPFGGGRRICPGLPMAIVQVPLILATLLHNFEWSLPNGEDLSKLDLNGRLGVTLQ
KYETLKLIPKRRLV
>CsNMCH2
MLIPSFSILLPSLLLLLPIFIILKHIKSPSSKLPLPPGPNPWPILGNLLQIGQNAHISITQFANIYG
PLISLKLGAQRLIVASSPAAATAVLKTQDRLLSARYIFQMTPDRALHDQHSLVFSPECGDQ
WKNLRSLCKVNLFTAKAIESQAILREKKMKELVKFLESKQGSVVEVKDFVFTSVFNTLSN
LIFSMDLLDFVGDGFKGIKGPFKKMMDLGLTPNLADFYPLLRRFDLQGLKNKADIYKNEV
DSLWGILIKERREIHCQQGSASNDFLDVLIQSGFDDQQINALIIELLSAGTDTTTTTVEWAM
SEILKDKDILNKVREEMKKVTKENRPIDESNISELQYLWRCVKETLRLHPPAPFLLPRLAP
MDCEVMGYSIPKDSMVFVNVWGIGRDPSVWEDSQTFNPQRFDVGCGNDNVDFKGYDY

RYIFAIWWRKKNLPWSSNGHCASSFDFSHINSQL
>CcNMCH1
MSFSLFLMSSFVLFTIRTYLKSEKGLPPGPRPWPIIGNLHQVGKNPHVSTAILAEKHGSLISL
RLGTQLLVVASSPEAATGILKTQDRFLSSRFIPNAFQSYLLPFALIWSADCDENWKSLRTLC
KTHMFSTKALESQSSLRERKLGEMLDFLCSKKDEVVNIEEVVFTTMFNTLSNILFGKDFLD
LRDRDGSASGLKEKLFKILANGLAPNVSDFFPMLDRWDLQGIKKEQLKHMKEIFSSWEDII
NDRRLMVVVKEEEQCFLDQLLENGFSNDQINILALELFTASTDTTTSTIEWAMAELIKNKK
AMFKLQEEIRNEIHSDTIFECELSKLSYLNACVKETLRLHPPTPFLLPHRAIQTCEVMNYTIP
RGSQVLVNIWAIGRNPKYWEDPLSFKPERFLNSKLDFKGQDFEFLPFGAGRRMCPGLFMG
INGVQSVLTYLILRFDWILPNDEDPLKIDMNEKFGVTLQKEKPLQLSFKHLK
>CcNMCH2
MDITSFLVHNNLSAPLFVLSTIFFLLLVVRLAKPTPSKNLPPGPPRLPIIGNLHQVGDRPHVS
TAKFANEYGPLISLRLGKQLLVVASSPDAXMEILKTQDRFLSSRVVPTAFQQASLIPHSLIW
SECNHTWKNLRTLCRTEMFSAKALESQSRLRDEKLGRLLDFLHGKQGQVINVEDVVFTTL
FNTLSSIIFARDFXDLKDERGSRDGLKESLHKIIEYGGIIKDLGSFFPIFERFDLQGIRKGTMK
QYXKTFAYWEDIVEERRALIXSSTWSSEQAQSFLDRLLENGFSNNQINQLVTELFVAGTNT
THTSVVWALTEFVRHKEVMSKIVDEIKREINSNKITDSQLSNLPYLQASIKEAMRLHPPVPL
LLPHMAAETCEVMNYTIPKNSKIFVNLWAMGRDPKVWDDPLSFKPERFMDSKLDLKGQD
FELLPFGSGRRMCPGMPSGIKSVQLVLASLIHEFDLILPNDVDPMKLNMNDKFGIALKMEE
PLKLIFKQKREPQXA
>BsCYP80A1
MDYIVGFVSISLVALLYFLLFKPKHTNLPPSPPAWPIVGHLPDLISKNSPPFLDYMSNIAQKY
GPLIHLKFGLHSSIFASTKEAAMEVLQTNDKVLSGRQPLPCFRIKPHIDYSILWSDSNSYWK
KGRKILHTEIFSQKMLQAQEKNRERVAGNLVNFIMTKVGDVVELRSWLFGCALNVLGHV
VFSKDVFEYSDQSDEVGMDKLIHGMLMTGGDFDVASYFPVLARFDLHGLKRKMDEQFK
LLIKIWEGEVLARRANRNPEPKDMLDVLIANDFNEHQINAMFMETFGPGSDTNSNIIEWAL
AQLIKNPDKLAKLREELDRVVGRSSTVKESHFSELPYLQACVKETMRLYPPISIMIPHRCM
ETCQVMGYTIPKGMDVHVNAHAIGRDPKDWKDPLKFQPERFLDSDIEYNGKQFQFIPFGS
GRRICPGRPLAVRIIPLVLASLVHAFGWELPDGVPNEKLDMEELFTLSLCMAKPLRVIPKVR
I
>AtCYP82C2
MDTSLFSLFVPILVFVFIALFKKSKKPKHVKAPAPSGAWPIIGHLHLLSGKEQLLYRTLGKM
ADQYGPAMSLRLGSSETFVVSSFEVAKDCFTVNDKALASRPITAAAKHMGYDCAVFGFAP
YSAFWREMRKIATLELLSNRRLQMLKHVRVSEISMVMQDLYSLWVKKGGSEPVMVDLKS
WLEDMSLNMMVRMVAGKRYFGGGSLSPEDAEEARQCRKGVANFFHLVGIFTVSDAFPKL
GWFDFQGHEKEMKQTGRELDVILERWIENHRQQRKVSGTKHNDSDFVDVMLSLAEQGK
FSHLQHDAITSIKSTCLALILGGSETSPSTLTWAISLLLNNKDMLKKAQDEIDIHVGRDRNV
EDSDIENLVYIQAIIKETLRLYPAGPLLGHREAIEDCTVAGYNVRRGTRMLVNVWKIQRDP
RVYMEPNEFRPERFITGEAKEFDVRGQNFELMPFGSGRRSCPGSSLAMQVLHLGLARFLQ
SFDVKTVMDMPVDMTESPGLTIPKATPLEILISPRLKEGLYV
>PsCYP82X1
MELFIKLPFIQPIPFSIILVTTVSIVLLYSVFFWVTDKKKKRKKAPNAAGAWPLIGHLRLLM
NDKEPLYRALGSMADKYGPAFNIRLGNQEVLVVSNWEMVKQCFGNQNDKLFSNRQTTL
AAKYMLNQTTSSGFAPYGPYWRELRKIMVQQLLSKQSLESWKHLKIKEMDASFSKLNEL

CNNNGTGTATLIRMDEWFAELTFNVIARNVFGYQSGGRSTALTNGDTESKGERYKKTLEE
ALHLMSIFAVSDIFPSLEWVDRLRGLIRNMKRFGDELNSIAGCLIEEHRQKRLQSVSKSDK
GVGDEQDFVDVLLSVAEKSQLPGDDPDLVIKSMILEIVSGGSETTSSTLTWALCLLLNHPH
VLKKAKEELDTHVGKDRHVEESDTPKLVYINAIIKESMRLYPNGAMLDRLALEECEVGGF
HVPAGGRLFVNVWKIQRDPSVWENPLEFKPERWFLSNGEKMDVDYKGHNHEFIPFGIGR
RMCAGMLWASEVIHLVLPRLIHGFDMKAASANGKVDMAEMAGMVICFKKTPLEVMVNP
RE
>PsCYP82X2
MKSLMMNKLLFLQRITDSPSTTIISTFIVTIISIVFLYTVLLIRTTKNKQKIAAPKASGAWPFI
GHLKLFMKQDTQFYRTLGTMSDKYGSVFTLRLGNQAILVVSNWEMVKECFTTNDKSFSN
RPSTLSTKYMLNDTNSVVFSPYGTYWREMRKILVQKLLISNQRSEALKNLKTKEIDNSFV
KLNDLCNNDVSGGGTKVRMDEWLADMMFNIIARITFGYQSGGGDAPGASTTSKNVERY
KKTLDEMFVVLATRFAVSDIFPSLEFIDRLRGLVKDMKILGDELNSIAGCFIEEHRQKRRES
LSSLLSLSNESVGDEQDFIDVLLSIMDQSRLPGDDPDFIIKIMILEAFAGGTDSLSATLTWVL
SLLLNHPNVLKRAREEIDRHVENGKQVEVSDIPKLGYIDAIIKETMRLYPVGALSERYTTE
ECEVGRFNVPAGTRLLVNIWKIHRDPSVWENPSDFQPERFLCSDKVGVDLYGQNYELIPFG
AGRRVCPAIVSSLQTMHYALARLIQGYEMKSASLDGKVNMEEMIAMSCHKMSPLEVIISP
REPRRS
